# Modelling chase-and-run migration in heterogeneous populations

**DOI:** 10.1007/s00285-019-01421-9

**Published:** 2019-08-29

**Authors:** A. Colombi, M. Scianna, K. J. Painter, L. Preziosi

**Affiliations:** 1 Department of Mathematical Sciences “G. L. Lagrange” - Excellence Department 2018-2022, Politecnico di Torino, Corso Duca degli Abruzzi, 24, 10129 Turin, Italy; 2 Department of Mathematics and Maxwell Institute for Mathematical Sciences, Heriot-Watt University, Edinburgh, Scotland EH14 4AS UK

**Keywords:** Neural crest, Invasion, Contact inhibition of locomotion, Agent-based model, 92C15, 92C17

## Abstract

Cell migration is crucial for many physiological and pathological processes. During embryogenesis, neural crest cells undergo coordinated epithelial to mesenchymal transformations and migrate towards various forming organs. Here we develop a computational model to understand how mutual interactions between migrating neural crest cells (NCs) and the surrounding population of placode cells (PCs) generate coordinated migration. According to experimental findings, we implement a minimal set of hypotheses, based on a coupling between chemotactic movement of NCs in response to a placode-secreted chemoattractant (Sdf1) and repulsion induced from contact inhibition of locomotion (CIL), triggered by heterotypic NC–PC contacts. This basic set of assumptions is able to semi-quantitatively recapitulate experimental observations of the characteristic multispecies phenomenon of “chase-and-run”, where the colony of NCs chases an evasive PC aggregate. The model further reproduces a number of in vitro manipulations, including full or partial disruption of NC chemotactic migration and selected mechanisms coordinating the CIL phenomenon. Finally, we provide various predictions based on altering other key components of the model mechanisms.

**Electronic supplementary material:**

The online version of this article (10.1007/s00285-019-01421-9) contains supplementary material, which is available to authorized users.

## Introduction

The neural crest is a transient structure, unique in embryonic vertebrates, that provides various neural derivatives which populate distinct tissues. It forms as a ribbon of ectoderm-derived cells and extends head to tail along the dorsal neural tube (Le Douarin and Kalcheim 1999; Theveneau and Mayor 2012). Notably, a controlled spatiotemporal epithelial-mesenchymal transition (EMT) allows detachment of its component cells, followed by their further migration and dispersion throughout the embryo as individuals, chains, clusters or sheets. Failure of neural crest cell (NC) dispersion/proliferation/differentiation can lead to various embryonic pathologies, collectively termed neurocristopathies (Watt and Trainor 2014). Beyond the critical function in early vertebrate life, neural crest dispersal offers a model system for studying the mechanisms underlying other cell invasion processes, such as those occurring during fibrosis or cancer development.

Various factors are implicated in NC migration and guidance, including homotypic and heterotypic cell-cell interactions, a combination of “go” and “no-go” factors in the extracellular matrix (ECM) and surrounding tissues that act to restrict NC migration along specific pathways, and long-range diffusible factors that provide chemotactic guidance (e.g., McLennan et al. 2010; Shellard and Mayor 2016; Theveneau et al. 2010; Mayor and Theveneau 2013; Tosney 2004; Twitty 1949). In particular, the study by Theveneau et al. (2010) has shown that *Xenopus laevis* NCs exhibit (positive) chemotaxis in the presence of gradients of the extracellular ligand Sdf1. Specifically, Sdf1 binds to the cell membrane receptor Cxcr4 and promotes intracellular Rac1, a key player in the activation and stabilisation of the cell motility structures (e.g., filopodia, pseudopodia) that lead to cell movement.

As remarked above, NC migration is also regulated by cell-to-cell contact interactions, which can be attractive, as in adhesion, or repulsive, as in contact inhibition of locomotion (CIL). CIL was first identified *in vitro* more than half a century ago (Abercrombie and Heaysman 1953), when the contact between two migrating fibroblasts was shown to lead to a transient arrest in their motion, a repolarisation and a subsequent reversal of migration heading. CIL therefore acts to promote cell repulsion and, intuitively, it could enhance dispersal. Current interest in CIL has been sparked by demonstrations that it also occurs *in vivo*, during dispersal of NCs in *Xenopus laevis* and zebrafish (Carmona-Fontaine et al. 2008; Theveneau et al. 2013). Further discoveries of its operation in cancer cell populations (Astin et al. 2010), developmental macrophages (Stramer et al. 2010) and neural cells (Villar-Cervino et al. 2013) have reinforced its relevance for migration and invasion processes.

*In vivo*, neural crest migration takes place in a highly heterogeneous environment where interactions with surrounding tissues or populations are inevitable. Recent studies of NC migration in *Xenopus laevis* indicate an active interplay between NCs and the epithelial-type “placode cells” (PCs) that initially lie adjacent to the neural crest (Theveneau et al. 2013). Such heterotypic interplay involves both long-range and contact-mediated interactions:

- PCs secrete the diffusible ligand Sdf1, which (as described above) acts as a chemoattractant for NCs and draws them towards PCs – the *chase* phase of the collective movement of the NC-PC system;
- direct contact between NCs and PCs then initiates a CIL response, invoking their movement away from each other – the *run* phase of the collective movement of the NC-PC system.

Cell-cell contacts are mediated through various signalling pathways, typically triggered by linkage of membrane-bound receptors on adjacent surfaces. In the case of NCs and PCs, cadherin family members (classically associated with adhesion) have been shown to play a significant role in their mutual dynamics. The initially attracting (adhesive) interactions that arise through N-cadherin–N-cadherin binding can subsequently give way to a repelling CIL response, mediated via a downstream signalling process. N-cadherin binding leads in fact to Rac1 downregulation, which in turn suppresses local cell membrane protrusions (Theveneau et al. 2013). Thus, protrusions become biased to the opposite end of the cell membrane from where the contact occurred and the individual cell is repolarised accordingly. Overall, N-cadherins therefore appear to generate both attracting (adhesion-type) and repelling (CIL-type) dynamics. While NCs solely express N-cadherins, placode cells also express E-cadherins, which generate stable homotypic E-cadherin bonds therefore promoting stable PC clustering.

**Fig. 1:**
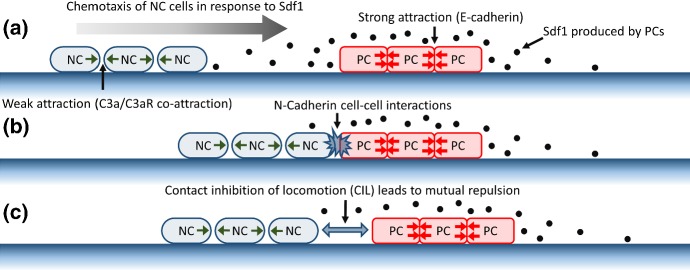
Schematic of “chase-and-run” dynamics. **a** NCs follow the chemoattractant gradient formed from Sdf1 producing PCs (Theveneau et al. 2013). PCs are tightly attached through E-cadherin binding (Theveneau et al. 2013), while NCs are more loosely connected via a combination of a C3a/C3aR co-attraction mechanism and NC-NC CIL responses (Carmona-Fontaine et al. 2008, 2011). **b** N-cadherin cell-cell interactions occur as the NC and PC populations collide, resulting in CIL in these two cell types (Theveneau et al. 2013). **c** The CIL response results in mutual repulsion. Overall, net movement of the two populations results

In the case of an *in vitro* aggregate of NCs juxtaposed against a similar aggregate of PCs, this “chase-and-run” process generates a net movement of the overall system, in which the NC cluster continuously chases PCs and is both repelled by and repels the PC population, see Fig. 1. *In vivo*, it has the potential to draw the NC population away from the neural crest and into the surrounding tissue, clearing a pathway through the PCs on route.

A number of theoretical works have addressed aspects of neural crest migration/collective migration: we refer to Schumacher et al. (2016) and Szabo and Mayor (2016) for specific reviews on the modelling of neural crest dynamics, and to Camley and Rappel (2017) for a review of collective cell migration models in general. With regard to CIL phenomena, one-to-one interactions between migrating and colliding NCs within quasi-one-dimensional geometries have been modelled in Kulawiak et al. (2016) and Merchant et al. (2018). The former developed a computational phase field model, explicitly accounting for cell shape and intracellular biochemistry. Consistent with experimental observations by Scarpa et al. (2013), different collision outcomes were observed (reversals, sticking and walk-past) according to the balance of different factors, such as adhesion. The model by Merchant et al. (2018) also investigated the outcome of collisions along 1D lines, in a model where each cell’s membrane is represented by a closed chain of elastic edges. Rac1/RhoA biochemistry was included via kinetic equations, with CIL and a NC-NC co-attraction process (mediated via the complement factor C3a and its receptor C3aR, see Carmona-Fontaine et al. 2011) incorporated through their action on kinetic terms. Sticking/reversal dynamics at a one to one level have also been investigated in Mayett et al. (2017) in an interacting elastic dimer model, where phase boundaries for the transition from clumping to chase-and-run were described for a NC colliding with a PC.

Extending to multicellular clusters, agent-based models have been analysed in Carmona-Fontaine et al. (2011) and Woods et al. (2014) to study the complementary roles of co-attraction and CIL in a population of homogeneous NC cells. Including both mechanisms allowed aggregates to migrate in an efficient, cohesive and directional manner. Along similar lines, a minimalistic stochastic particle model featuring a chemoattractant-regulated CIL process was shown to be sufficient to explain efficient cluster migration (Camley et al. 2016a), while extensions generated more sophisticated dynamics such as particle rotations about a core (Camley et al. 2016b). In Szabo et al. (2016), the ECM molecule versican was shown to display a complex spatio-temporal pattern of tissue expression which, when combined with its capacity to restrict NC migration, was supposed to channel NC invasion along specific routes. A Cellular Potts Model approach that incorporated CIL and co-attraction between NCs alongside versican-mediated inhibition of migration helped verify this hypothesis. The above described model by Merchant et al. (2018) has also been applied to explore NC cluster migration, demonstrating how these two mechanisms generate spontaneous and efficient collective migration.

Beyond these studies targeted at CIL/chase-and-run dynamics, further theoretical studies have addressed NC migration in other systems. Cranial neural crest cell migration in chicken embryos has been explored by Kulesa and colleagues (McLennan et al. 2012, 2015a, b), where agent-based models were used to understand the role of “trailblazer” cells in forging a path through the tissue. Specifically, a trailblazer population undergoes chemotaxis in response to self-created gradients of an externally-produced attractant, while follower cells simply chase the leaders. Other neural crest focussed models have been developed to describe invasion of mouse enteric neural crest cells (Cheeseman et al. 2014; Landman et al. 2007, 2011; Simpson et al. 2007) and mouse melanocyte cells (Mort et al. 2016), although in those systems cell migration is augmented with significant proliferation that helps drive the dispersal process.

The principal aim of our work is to model “chase-and-run” collective behaviour observed *in vitro* in multicellular NC-PC systems. Within this scenario, any cellular growth, birth or death processes appear to be minimal, allowing us to solely focus on the interactions that drive coordinated movement. We propose a hybrid multiscale approach, in which cells are individually described as microscopic/discrete interacting particles and PC-produced Sdf1 is represented by a continuous concentration distribution. Moving beyond the studies described above, we specifically consider chase and run within multicellular and heterogeneous clusters, composed from both NCs and PCs. Further, we model the dynamics of the extracellular chemical substance (Sdf1) via an explicit evolution equation that describes its spatiotemporal dynamics. For computationally manageability and limiting the dimensionality of the parameter set, we formulate a minimalistic set of interactions in order to understand the basic requirements necessary for “chase-and-run” dynamics. *In silico* experiments reveal the model’s capacity to replicate features observed *in vitro*, both in the case of one cell-to-one cell and cluster-to-cluster interactions. Further, our model reproduces a number of experimental perturbations that target key mechanisms involved during “chase-and-run” dynamics and can be used to make a number of testable predictions.

## The model

The overall theoretical framework consists of a proper set of ordinary differential equations (ODEs) for the cell dynamics and a reaction-diffusion (RD) law for Sdf1 kinetics. Our general system involves $n_P$ placode cells and $n_N$ neural crest cells, restricted so that they migrate across a planar domain $\varOmega \subset {\mathbb {R}}^2$ (e.g., an experimental Petri dish as used for *in vitro* assays). Regardless of phenotype, each cell is represented by a discrete pointwise agent and can be identified by its position in space, say ${\mathbf {x}}_{i}^{{\alpha }}(t)\in \varOmega$ with ${\alpha }\in \left\{ N,P\right\}$, $i=1,\dots ,n_{\alpha }$ and $t\in {\mathbb {R}}_+$. However, physical cell sizes are taken into account via defining the evolution equations for cell distribution and movement. Specific scenarios, such as heterotypic interactions between single cells or homotypic aggregates, follow through tuning the sizes of $n_N$ and $n_P$. In the rest of this section we motivate and define the underlying equations upon which our model is formulated, introducing a set of parameters in the process. For reference, these parameters are subsequently summarised in Table 1 while Sect. 3.1 lays out our parameter estimation. Specifically, we highlight those estimated from source values and those obtained via a data fitting process.

### Cell dynamics

Cell dynamics are described by a first-order ODE system, which is derived from a general second-order particle model following a standard set of simplifying biological considerations. First, cells move in environments characterized by very low Reynolds numbers (Van Liedekerke et al. 2015; Odell et al. 1981), where ballistic locomotion is only briefly maintained and straight-line displacements are shorter than the typical cell dimension. Consequently, we can neglect inertial effects and adopt a first-order model in which cell velocity, rather than acceleration, is proportional to the acting forces. This relation, termed as overdamped force-velocity response, lies at the heart of numerous discrete/ individual-based-model (IBM) approaches (see Drasdo 2003; Scianna and Preziosi 2012 and references therein for details) and demands that cell dynamics are driven by cell-cell friction and cell-substrate friction. For the planar domain considered here, cell-substrate friction is particularly relevant since the cell will have a larger contact area with the substrate compared to neighbouring cells. We therefore also neglect cell-cell friction terms so that, as a first approximation, cell dynamics can be described by directly postulating cell velocity contributions and including cell-substrate friction coefficients in their characteristic parameters (see Carrillo et al. 2018 for further details).

**Table 1:** Default model parameter set for simulations (where “d.f.” states for “data fitting”)

Comp.	Def.	Par. & Val.	References
Cell size	Perinuclear region	$d_r = 20~\upmu \hbox {m}$	Theveneau et al. (2013)
	Body diameter	$d_c = 30~\upmu \hbox {m}$	Theveneau et al. (2013)
	Filopodia extension	$d_a = 60~\upmu \hbox {m}$	Theveneau et al. (2013)
${\mathbf {v}}_{i,\,\text {res}}^{{\alpha }}$	Repulsion strength	$F_r^{{\alpha }{\beta }} = F_r = 1~\upmu \hbox {m}\,\hbox {s}^{-1}$	
		$\quad \quad {\alpha },{\beta }\in \{N,\,P\}$	d.f.
${\mathbf {v}}_{i,\,\text {adh}}^{{\alpha }}$	NC-NC adhesion	$F^{NN}_a=0.002~\upmu \hbox {m}\,\hbox {s}^{-1}$	d.f.
	NC-PC adhesion	$$F^{NP}_a=F^{PN}_a = $$	
		$\quad \quad = 0.002~\upmu \hbox {m}\,\hbox {s}^{-1}$	d.f.
	PC-PC adhesion	$F^{PP}_a = 0.008~\upmu \hbox {m}\,\hbox {s}^{-1}$	d.f.
${\mathbf {v}}_{i,\,\text {CIL}}^{{\alpha }{\beta }}$	Decay times	$\tau _{NP}=\tau _{PN} = 6\,\mathrm {min}$	Scarpa et al. (2013)
			Theveneau et al. (2013)
	Instantaneous push on NC	$\omega _{NP}=5000$	d.f. on Scarpa et al. (2013)
			Theveneau et al. (2013)
	Instantaneous push on PC	$\omega _{PN}=2500$	d.f. on Scarpa et al. (2013)
			Theveneau et al. (2013)
${\mathbf {v}}_{i,\,\text {chem}}^{N}$	Maximal cell speed	$v_{\text {max}} = 0.0333~\upmu \hbox {m}\,\hbox {s}^{-1}$	Scarpa et al. (2013)
			Theveneau et al. (2013)
	Chemotactic sensitivity	$\chi _{N}=500~\upmu \hbox {m}^2~\upmu \hbox {M}^{-1}\,\hbox {s}^{-1}$	Colombi et al. (2015)
${\mathbf {v}}_{i,\,\text {rand}}^{{\alpha }}$	NC random motility	$v_{\text {rand}}^{N} = 0.6~\upmu \hbox {m}\,\hbox {s}^{-1}$	d.f. on Theveneau et al. (2013)
	PC random motility	$v_{\text {rand}}^{P} = 0.05~\upmu \hbox {m}\,\hbox {s}^{-1}$	d.f. on Theveneau et al. (2013)
Sdf1	Diffusion coefficient	$D = 13.3~\upmu \hbox {m}^2\,\hbox {s}^{-1}$	Szabo et al. (2016)
	Decay rate	$\varepsilon = 0.0004\,\hbox {s}^{-1}$	Szabo et al. (2016)
	Production rate	$c_0 = 0.027~\upmu \hbox {M}\,\hbox {s}^{-1}$	Szabo et al. (2016)

**Fig. 2:**
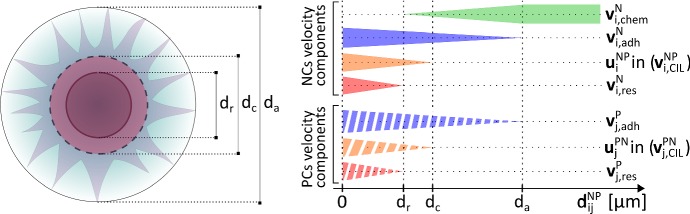
Left panel: representation of cell morphology (approximately to scale, see Table 1 for values of $d_r, d_c, d_a$). NCs and PCs do not reveal significant differences in morphology and dimensions and hence, regardless of cell lineage: $d_r$ denotes the mean perinuclear region diameter; $d_c$ is the mean cell body diameter; $d_a$ describes the maximal extension of the motility structures that protrude from the cell surface (e.g., filopodia, lamellipodia, pseudopodia). Right panel: conceptual representation of velocity contributions, given in Eq. (1), in the simplified representative case of a heterotypic two-cell system, i.e., composed of the generic *j*-th PC and the generic *i*-th NC cell. Each bar indicates when a given velocity component is activated according to $d_{ij}^{NP}$. Specifically, solid bars refer to the dynamics of the *i*-th NC, while dashed bars represent velocity components affecting the *j*-th PC. In both instances, bar thickness denotes how their magnitude varies with cell-cell distance $d_{ij}^{NP}$

The velocity of each cell is then determined by a sum of contributions that stem from its long and short range interactions with other agents, which may be directed or mediated by chemical signalling. Motivated by the experimental literature (Steventon et al. 2014; Szabo and Mayor 2015; Theveneau et al. 2013), the hypotheses at the heart of the model are as follows.

1. Each cell has an intrinsic resistance to compression, mainly due to the stiffness of its nuclear and perinuclear region. This force acts to repel two cells separated by distances smaller than $d_r$ (the mean perinuclear region diameter), see Fig. 2.
2. Two cells can form adhesive bonds through the activity of transmembrane proteins. In particular, N-cadherins regulate both heterotypic (NC-PC) contacts and homotypic (NC-NC or PC-PC) adhesive interactions, while homotypic PC-PC bonds are further augmented by E-cadherins. The force generated through adhesive bonds acts to attract two cells up to a distance $d_a$, which denotes the maximal extension of protruding motility structures (lamellipodia, filopodia etc.) from the cell’s centre, see Fig. 2.
3. Heterotypic N-cadherin bonds trigger a Rac1-dependent contact inhibition of locomotion (CIL) response in pairs of interacting cells, whereby adhesive complexes collapse, cytoskeletons repolarise and individuals move away from each other (the *run* phase of the process). Specifically, we assume the CIL response begins when the distance between two interacting cells falls below a critical value, $d_c$, which is a measure of the cell diameter, see Fig. 2. The run phase duration is characterised by timescales $\tau _{{\alpha }{\beta }}$ for ${\alpha }{\beta }\in \left\{ NP,PN\right\}$.
4. NCs chemotactically migrate in response to PC-produced Sdf1. This directional locomotion (the *chase* phase of the process) is subsequently downregulated once NC-PC adhesive complexes form, as well as once the NC is compressed by surrounding cells. Due to the extracellular and diffusible nature of Sdf1, chemotaxis is assumed to operate over arbitrary distances (although with a response magnitude varying with the detected attractant gradient).
5. Each cell, regardless of type, shows innate random wandering. This Brownian crawling is taken to be more pronounced for NCs over PCs, as observed by comparing the dynamics of homotypic aggregates (see in Theveneau et al. 2013, Fig. 2(a)–(b)).

The overall system of ODEs regulating cell behaviour can therefore be written as follows:

1 $$\begin{aligned} {\left\{ \begin{array}{ll} \dfrac{d{\mathbf {x}}_{i}^{N}(t)}{dt} &{}= \underbrace{{\mathbf {v}}_{i,\,\text {res}}^{N}(t)}_{\text {hp.}\;\text {1}} +\underbrace{{\mathbf {v}}_{i,\,\text {adh}}^{N}(t)}_{\text {hp.}\;\text {2}} +\underbrace{{\mathbf {v}}_{i,\,\text {CIL}}^{NP}(t)}_{\text {hp.}\;\text {3}} +\underbrace{{\mathbf {v}}_{i,\,\text {chem}}^{N}(t)}_{\text {hp.}\;\text {4}} +\underbrace{{\mathbf {v}}_{i,\,\text {rand}}^{N}(t)}_{\text {hp.}\;\text {5}},\\ &{}\qquad \qquad \quad \,\, i=1,\dots ,n_N;\\ \dfrac{d{\mathbf {x}}_{j}^{P}(t)}{dt}&{}= \underbrace{{\mathbf {v}}_{j,\,\text {res}}^{P}(t)}_{\text {hp.}\;\text {1}} +\underbrace{{\mathbf {v}}_{j,\,\text {adh}}^{P}(t)}_{\text {hp.}\;\text {2}} +\underbrace{{\mathbf {v}}_{j,\,\text {CIL}}^{PN}(t)}_{\text {hp.}\;\text {3}} +\underbrace{{\mathbf {v}}_{j,\,\text {rand}}^{P}(t)}_{\text {hp.}\;\text {5}},\\ &{}\qquad \qquad \quad \,\, j=1,\dots ,n_P. \end{array}\right. } \end{aligned}$$

Let us now describe in more detail the cell velocity contributions introduced above.

**Fig. 3:**
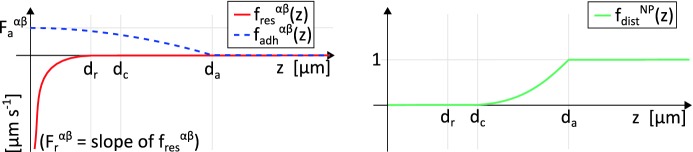
Left panel: representation of the functions used to describe cell resistance to compression ($f^{{\alpha }{\beta }}_{\text {res}}$, solid red curve) and adhesion ($f^{{\alpha }{\beta }}_{\text {adh}}$, dashed blue curve) for any ${\alpha }{\beta }$ pair with ${\alpha },{\beta }\in \left\{ N,P\right\}$. Repulsive interactions, defined in Eq. (4), affect the dynamics of a cell of type ${\alpha }$ when its distance from a cell of type ${\beta }$ is lower than its nuclear dimension, denoted by $d_r$. As defined in Eq. (6), adhesive interactions arise when the relative distance between the two cells is lower than the maximal extension of plasma membrane motility structures, measured by $d_a$. Right panel: representation of the function used to describe NC chemotactic migration ($f_{\text {dist}}^{NP}$). As defined in Eq. (10), any chemotactic response of the generic NC starts to be downregulated once it undergoes adhesive interactions with at least one PC (when $z\le d_a$), becoming negligible when the two cells are tightly packed ($z\le d_c$) (color figure online)

*Cell resistance to compression* The terms ${\mathbf {v}}^{N}_{i,\,\text {res}}$ and ${\mathbf {v}}^{P}_{j,\, \text {res}}$ reproduce short-range intercellular repulsion, activated when the body of a cell is compressed by other individuals. In particular, it is natural to assume that for each individual this velocity contribution: (i) results from the superposition of its pairwise interactions; (ii) is strictly dependent on the relative distance between the cells involved (i.e., metric interactions); and (iii) is directed along the unit vector connecting their positions. Hence, we can write

2 $$\begin{aligned} \left. \begin{array}{lll} \mathbf{v}_{i,\,\text {res}}^{N}(t) &{}=&{} \displaystyle \sum ^{n_N}_{k=1, k\ne i} f^{NN}_{\text {res}}(d^{NN}_{ik})\,\hat{\mathbf{r}}^{NN}_{ik} + \sum ^{n_P}_{h=1} f^{NP}_{\text {res}}(d^{NP}_{ih})\,\hat{\mathbf{r}}^{NP}_{ih}, \quad i=1,\dots ,n_N,\\ \mathbf{v}_{j,\,\text {res}}^{P}(t) &{}=&{} \displaystyle \sum ^{n_P}_{ h=1, h\ne j} f^{PP}_{\text {res}}(d^{PP}_{jh})\,\hat{\mathbf{r}}^{PP}_{jh}+ \sum ^{n_N}_{k=1} f^{PN}_{\text {res}}(d^{PN}_{jk})\,\hat{\mathbf{r}}^{PN}_{jk}, \quad j=1,\dots ,n_P, \end{array} \right. \end{aligned}$$

where $f_{\text {res}}^{{\alpha }{\beta }}:{\mathbb {R}}_{+}\longmapsto {\mathbb {R}}_{-}$, with ${\alpha },{\beta }\in \left\{ N,P\right\}$, are interaction kernels determining the intensity of cell repulsive behaviour. Further, we define

3 $$\begin{aligned} d^{{\alpha }{\beta }}_{ij}=|\mathbf{x}_{j}^{\beta }-{\mathbf{x}_{i}^{\alpha }}| \quad \mathrm{and}\quad \hat{\mathbf{r}}^{{\alpha }{\beta }}_{ij}= \dfrac{\mathbf{x}_j^{\beta }-\mathbf{x}_i^{\alpha }}{|\mathbf{x}_j^{\beta }-\mathbf{x}_i^{\alpha }|}\,, \end{aligned}$$

for $i= 1,\dots ,n_{{\alpha }}$, $j=1,\dots ,n_{{\beta }}$, and ${\alpha },{\beta }\in \left\{ N,P\right\}$, where $|\cdot |$ denotes the Euclidean norm. Trivially, $d^{{\alpha }{\beta }}_{ij}$ is invariant under changes to the order of superscripts and subscripts, while $\hat{\mathbf{r}}^{{\alpha }{\beta }}_{ij}$ undergoes sign change with the order of subscripts. In principle, there is a wide range of choice for the form of functions $f_{\text {res}}^{{\alpha }{\beta }}$, however, certain biological observations aid our selection. First, any contact-dependent interaction vanishes when the individuals involved are too distant. Moreover, cell resistance to compression typically increases as the distance between the interacting cells decreases. Hence, taking advantage of existing literature (see in particular Colombi et al. 2015, 2017; Scianna and Colombi 2017), a plausible option is

4 $$\begin{aligned} f_{\text {res}}^{{\alpha }{\beta }}(z)= {\left\{ \begin{array}{ll} F_r^{{\alpha }{\beta }}\left( 1 - \dfrac{d_r}{z}\right) , &{} \text {if } z\le d_r\,;\\ 0, &{} \text {otherwise}\,, \end{array}\right. } \end{aligned}$$

with, as previously stated, $d_r$ being the mean diameter of the cell perinuclear region (see Fig. 2) and taken to be equal for both cell lineages. $F_r^{{\alpha }{\beta }}>0$, with ${\alpha },{\beta }\in \left\{ N,P\right\}$, instead determines the slope of the repulsive kernel, which can be related both to the intrinsic stiffness of the interacting cells and to the compression force (thereby depending on the type of individuals involved). A plot of the repulsive kernel $f_{\text {res}}^{{\alpha }{\beta }}$ is shown in the left panel of Fig. 3 (solid red curve).

*Cell-cell adhesion* The terms ${\mathbf {v}}^{N}_{i,\, \text {adh}}$ and ${\mathbf {v}}^{P}_{j,\, \text {adh}}$ describe homotypic and heterotypic adhesive dynamics due to the activation of selected cadherin molecules. Their form is the sum of pairwise interactions analogous to the repulsive counterpart, i.e.,

5 $$\begin{aligned} \left. \begin{array}{lll} \mathbf{v}_{i,\,\text {adh}}^{N}(t) &{}=&{} \displaystyle \sum ^{n_N}_{k=1, k\ne i} f^{NN}_{\text {adh}}(d^{NN}_{ik})\,\hat{\mathbf{r}}^{NN}_{ik}\, + \sum ^{n_P}_{h=1} f^{NP}_{\text {adh}}(d^{NP}_{ih})\,\hat{\mathbf{r}}^{NP}_{ih}\,, \quad i=1,\dots , n_N,\\ \mathbf{v}_{j,\,\text {adh}}^{P}(t) &{}=&{} \displaystyle \sum ^{n_P}_{h=1, h\ne j} f^{PP}_{\text {adh}}(d^{PP}_{jh})\,\hat{\mathbf{r}}^{PP}_{jh}\, + \sum ^{n_N}_{k=1} f^{PN}_{\text {adh}}(d^{PN}_{jk})\,\hat{\mathbf{r}}^{PN}_{jk}\,, \quad j=1,\dots , n_P\,, \end{array} \right. \end{aligned}$$

where $f_{\text {adh}}^{{\alpha }{\beta }}:{\mathbb {R}}_{+}\longmapsto {\mathbb {R}}_{+}$ (with ${\alpha },{\beta }\in \left\{ N,P\right\}$) are the corresponding kernels. The explicit form of $f_{\text {adh}}^{{\alpha }{\beta }}$ can be established by assuming that a pair of cells must be sufficiently close to adhere. Specifically, we assume adhesions start to form if their mutual distance is below the maximal extension of membrane protrusions, $d_a$ (see Fig. 2). Further, adhesion increases as the contact surface between two individuals grows, which follow as the mutual distance drops. Taking all of these considerations into account, for any couple ${\alpha }{\beta }$ with ${\alpha },{\beta }\in \left\{ N,P\right\}$, we opt for the following family of functions:

6 $$\begin{aligned} f_{\text {adh}}^{{\alpha }{\beta }}(z)= {\left\{ \begin{array}{ll} F_a^{{\alpha }{\beta }}\,\left( 1 - \dfrac{z^2}{d_a^2}\right) , &{} \text {if } z \le d_a\,; \\ 0, &{} \text {otherwise}\,, \end{array}\right. } \end{aligned}$$

where $F_a^{{\alpha }{\beta }}>0$ is a measure of the maximal adhesive stimulus, occurring for highly overlapping cells (dashed blue curve in the left panel of Fig. 3).

*CIL velocity component* The velocity contributions ${\mathbf {v}}^{NP}_{i,\, \text {CIL}}$ and ${\mathbf {v}}^{PN}_{j,\, \text {CIL}}$ describe heterotypic CIL responses, resulting in mutual cell repulsion. To translate this mechanism into mathematical terms, we first note that the CIL response activates when a neural crest, say *i*, and a placodal cell, say *j*, come into contact. In other words, it occurs when their relative distance $d^{NP}_{ij}=d^{PN}_{ji}$ falls below the critical value $d_c$ which, as previously explained, gives a measure of the cell diameter (see again Fig. 2), assumed equal for both cell lineages (Theveneau et al. 2013). The durations of the subsequent phases of reflected directional migration are characterised by decay times, say $\tau _{NP}$ for NCs and $\tau _{PN}$ for PCs. The CIL velocity components ${\mathbf {v}}^{NP}_{i,\,\text {CIL}}$ and ${\mathbf {v}}^{PN}_{j,\,\text {CIL}}$ are therefore assumed to evolve according to:

7 $$\begin{aligned} {\left\{ \begin{array}{ll} \dfrac{d\mathbf{v}^{NP}_{i,\,\text {CIL}}}{dt}(t) = \dfrac{1}{\tau _{NP}} \left( \omega _{NP}\,F_a^{NP}\,\dfrac{\mathbf{u}^{NP}_{i}(t)}{|\mathbf{u}^{NP}_{i}(t)|} - \mathbf{v}^{NP}_{i,\,\text {CIL}}(t)\right) \,, \quad i=1,\dots ,n_N;\\ \dfrac{d\mathbf{v}^{PN}_{j,\,\text {CIL}}}{dt}(t) = \dfrac{1}{\tau _{PN}} \left( \omega _{PN}\,F_a^{PN}\,\dfrac{\mathbf{u}^{PN}_{j}(t)}{|\mathbf{u}^{PN}_{j}(t)|} - \mathbf{v}^{PN}_{j,\,\text {CIL}}(t)\right) \,, \quad j=1,\dots ,n_P. \end{array}\right. } \end{aligned}$$

In particular, $\omega _{NP}$ and $\omega _{PN}$ quantify the size of the instantaneous push, which could (amongst other factors) be dictated by both the number of heterotypic N-cadherin adhesive bonds and the level of activation of the downstream intracellular cascade. $\mathbf{u}^{NP}_{i}$ and $\mathbf{u}^{PN}_{j}$ define the subsequent direction of the CIL-induced migration that follows re-polarisation. For a two cell scenario, these are aligned with the unit vector $\hat{\mathbf{r}}_{11}^{NP}$: in particular, $\mathbf{u}^{NP}_{1} = -\hat{\mathbf{r}}_{11}^{NP}$ and $\mathbf{u}^{PN}_{1} = -\hat{\mathbf{r}}_{11}^{PN}$. In a multicellular scenario, we have instead to account for the possibility of multiple simultaneous heterotypic contacts. Specifically, for $h=1,\dots ,n_{{\alpha }}$ with ${\alpha },{\beta }\in \left\{ N,P\right\}$ and ${\alpha }\ne {\beta }$, $\mathbf{u}^{{\alpha }{\beta }}_{h}$ is taken to be the weighted sum of the unit vectors $\hat{\mathbf{r}}_{hk}^{{\alpha }{\beta }}$ that connect the *h*-th cell of type ${\alpha }$ with the surrounding *k*-th particle of type ${\beta }$:

8 $$\begin{aligned} \mathbf{u}^{{\alpha }{\beta }}_{h}(t) = - \sum _{k = 1}^{n_{\beta }} \left( 1 - \dfrac{d^{{\alpha }{\beta }}_{hk}}{d_c}\right) _{+} \hat{\mathbf{r}}^{{\alpha }{\beta }}_{hk}\,, \end{aligned}$$

where $(\,\cdot \,)_+=\frac{(\,\cdot \,)+|\,\cdot \,|}{2}$, and $d^{{\alpha }{\beta }}_{hk}$ and $\hat{\mathbf{r}}^{{\alpha }{\beta }}_{hk}$ have been defined in Eq. (3). The positive part function $(\,\cdot \,)_+$ is necessary in Eq. (8) to ensure that the CIL response is triggered only when $d_{hk}^{\alpha \beta }\le d_c$. The above equation implements the assumption that the closest agents are those that generate the largest contribution when establishing the CIL velocity direction component: this is a reasonable hypothesis, since nearest individuals are likely to form a larger number of more extended cadherin complexes, thereby triggering a more significant CIL response.

The adhesive coefficients $F_a^{NP}$ and $F_a^{PN}$ included in Eq. (7) account for the fact that the intracellular cascade underlying CIL is mediated through N-cadherin bonds. This allows us to then implement selective experimental manipulations, such as the inhibition of N-cadherin adhesive complex bond formation (by setting $F_a^{NP}=F_a^{PN} = 0~\upmu {\hbox {m}\,\hbox {s}}^{-1}$) or the disruption of the downstream intracellular cascade regulating cell re-polarisation (by setting $\omega _{NP} =\omega _{PN} =0$).

Finally, we assume that for the duration of a CIL contribution between the *i*-th NC and the *j*-th PC, i.e., ${\mathbf {v}}^{NP}_{i,\text {CIL}}$ and ${\mathbf {v}}^{PN}_{j,\text {CIL}}$, adhesive interactions between the two individuals are neglected (i.e., ${\mathbf {v}}^{N}_{i,\text {adh}}={\mathbf {v}}^{P}_{j,\text {adh}}=0$), even given low intercellular distances (i.e., $d^{NP}_{ij}=d^{PN}_{ji}< d_a$). From a biological point of view, this is reasonable given that CIL triggers the collapse of the adhesive complexes in addition to re-polarization of the cell cytoskeleton, see Steventon et al. (2014), Szabo and Mayor (2015), Theveneau et al. (2013).

*Chemotaxis velocity component* The term ${\mathbf {v}}^{N}_{i,\,\text {chem}}$ implements the chemotactic migration of the *i*-th NC towards higher concentrations of the PC-produced Sdf1. With $c(\mathbf{x },t)$ denoting the concentration of Sdf1, we set

9 $$\begin{aligned} {\mathbf {v}}^{N}_{i,\,\text {chem}}(t) = f_{\text {dist}}^{NP}(d_{i,\,\text {min}}^{NP}(t))\; \min \left\{ v_{\text {max}},\,\chi _{N}\, |\nabla c(\mathbf{x}_i^N(t),t)|\right\} \, \dfrac{\nabla c(\mathbf{x}_i^N(t),t)}{|\nabla c(\mathbf{x}_i^N(t),t)|}\,,\nonumber \\ \end{aligned}$$

where $d_{i,\,\text {min}}^{NP} := \min _{k=1,\dots ,n_P} d_{ik}^{NP}$. The function $f_{\text {dist}}^{NP}:{\mathbb {R}}_+\rightarrow [0,1]$ is defined as follows

10 $$\begin{aligned} f_{\text {dist}}^{NP}(z) = {\left\{ \begin{array}{ll} 0, &{}\text {if } 0< z \le d_c;\\ \dfrac{(z-d_c)^2}{(d_a-d_c)^2}, &{} \text {if } d_c < z \le d_a; \\ 1, &{} \text {if } z > d_a, \end{array}\right. } \end{aligned}$$

as represented in Fig. 3 (right panel). The above law reduces chemotactic movement of NCs as they start to undergo adhesive interactions with at least one PC (i.e., if $d_{i,\,\text {min}}^{NP}\le d_a$) and ensures it becomes negligible for close contact (i.e., if $d_{i,\,\text {min}}^{NP}\le d_c$). Biologically, this could reflect the intracellular integration of chemotactic/adhesion pathways, where the positive promotion of Rac1 (and hence cell protrusions) by chemotaxis is negated following N-cadherin binding (Theveneau et al. 2013). In Eq. (9), $\chi _{N}$ represents a chemotactic sensitivity parameter, e.g., the functionality of Sdf1 membrane receptors Cxcr4, and is taken to be equal for all NCs. To avoid unrealistic cell speeds, ${\mathbf {v}}^{N}_{i,\,\text {chem}}$ is capped by parameter $v_{\text {max}}$, denoting the measured maximal speed of NCs.

*Random velocity component* The terms ${\mathbf {v}}_{i,\,\text {rand}}^{N}$ and ${\mathbf {v}}_{j,\,\text {rand}}^{P}$ account for the isotropic fluctuations that impact on cell trajectories, as observed in biological experiments by Theveneau et al. (2013). Specifically,

11 $$\begin{aligned} \left. \begin{array}{lll} \mathbf{v}_{i,\,\text {rand}}^{N}(t) &{}=&{} v_{\text {rand}}^{N}\, \begin{pmatrix} \cos (\theta ^{N}_{i}(t))\\ \sin (\theta ^{N}_{i}(t)) \end{pmatrix} \quad i = 1,\dots ,n_N\,,\\ \mathbf{v}_{j,\,\text {rand}}^{P}(t) &{}=&{} v_{\text {rand}}^{P}\, \begin{pmatrix} \cos (\theta ^{P}_{j}(t))\\ \sin (\theta ^{P}_{j}(t)) \end{pmatrix} \quad j = 1,\dots ,n_P\,, \end{array} \right. \end{aligned}$$

where $\theta _h^{{\alpha }}(t)$, with $\alpha \in \{N, P\}$, is a random angle uniformly distributed over $[0,2\pi )$. $v^{N}_{\text {rand}}$ and $v^{P}_{\text {rand}}$ are constant speeds, estimated accounting for observations in *in vitro* assays in Theveneau et al. (2013). Of course, while other more sophisticated models for incorporating random movement could be considered, the lack of any detailed biological data motivates us to consider the above form for its simplicity.

### Sdf1 kinetics

In addition to Eq. (1), we require an equation for Sdf1 kinetics. In particular, the chemical substance is assumed to be secreted (at a constant rate) by all placode cells, to diffuse through the surrounding environment and to degrade at a constant rate. Its spatiotemporal evolution can therefore be modelled by the following classical reaction-diffusion (RD) equation:

12 $$\begin{aligned} \dfrac{\partial c}{\partial t}(\mathbf{x },t) = D\,\varDelta c(\mathbf{x },t) - \varepsilon \,c(\mathbf{x },t) +c_{0}\,\sum _{j=1}^{n_P}\delta _{{\mathbf {x}}^{P}_{j}(t)}(\mathbf{x },t)\,, \end{aligned}$$

where *D* is a homogeneous diffusion coefficient and $\varepsilon$ and $c_0$ are respectively the decay and production rates. The functions $\delta _{{\mathbf {x}}^{P}_{j}(t)}$ describe point sources, centred on the PC positions.

## Results

### Simulation details and parametrisation

All numerical simulations are performed on a bounded square domain of dimensions $700~\upmu \hbox {m}\times 700~\upmu \hbox {m}$, based on the biological images reported in Theveneau et al. (2013). If a cell *i* touches the boundary $\partial \varOmega$ at time $t\in [0,T_{\text {max}}]$, it is assumed to stop (i.e., ${\mathbf {v}}_i^{{\alpha }}(t)={\mathbf {0}}$); Sdf1 is taken to be absorbed at the boundary, i.e., $c(\mathbf{x},t)=0$, $\forall \,\mathbf{x}\in \partial \varOmega$ and $\forall \,t\in {\mathbb {R}}_+$. At the start of each simulation we assume zero Sdf1 (i.e., $c({\mathbf {x}},0) = 0$, $\forall \ {\mathbf {x}}\in \varOmega$) and neglect CIL-related velocity components (i.e., $\mathbf{v }^{NP}_{i,\text {CIL}}(0) = \mathbf{v }^{PN}_{j,\text {CIL}}(0) = \mathbf{0 }$, $\forall \ i=1,\dots ,n_N$ and $j=1,\dots ,n_P$). Each *in silico* experiment runs until a characteristic observation time, $T_{\text {max}}$, set at $300~\mathrm {min} = 5\mathrm {h}$. Initial cell distributions are specified for each simulation suite.

While many parameters can be directly estimated from biological data, some have no clear experimental correspondence. Their estimate is, rather, based on a process of sensitivity analysis supported by empirical measurements and observations. Default parameters used in simulations are listed in Table 1, with those obtained via data fitting indicated. Precise details for parameter estimation are given below.

*Cell sizes* From the biological literature, NCs and PCs appear to have similar morphology. In particular, an analysis of the experimental images in Theveneau et al. (2013) allows us to fix a common diameter of the perinuclear region $d_r=20~\upmu \hbox {m}$ and a maximal filopodia extension at $30~\upmu \hbox {m}$, so that $d_a=60~\upmu \hbox {m}$. The mean body diameter of fully adherent cells ($d_c$) is then set equal to the intermediate value, i.e., $d_c = 30~\upmu \hbox {m}$.

*Parameters related to Sdf1 kinetics and chemotactic velocity components* Values for coefficients relevant for Sdf1 kinetics in Eq. (12) are set according to those used in Szabo et al. (2016): $D = 13.3~\upmu \hbox {m}^2\, \hbox {s}^{-1}$, $\varepsilon = 0.0004\,\hbox {s}^{-1}$ and $c_0 = 0.027~\upmu \hbox {M}\,\hbox {s}^{-1}$. The maximal chemotaxis velocity $v_{\text {max}}$ is instead set at $120~\upmu \hbox {m} \mathrm {h^{-1}} = 0.0333~\upmu \hbox {m}\,\hbox {s}^{-1}$, according to the biological measurements reported in Theveneau et al. (2010). Finally, the chemotactic sensitivity coefficient $\chi _N$ is set at $500~\upmu \mathrm {m^2~\upmu \hbox {M}^{-1} \mathrm {s}^{-1}}$, on the basis of Colombi et al. (2015), where chemotaxis is stimulated by a chemical factor (VEGF) with a similar diffusion coefficient. Model responses to independent variations of $c_0$ and $\chi _N$ will be described in the following section.

*Parameters related to cell resistance to compression and cell-cell adhesion* As far as we are aware, there is no evidence that placode and neural crest cells have a different nuclear stiffness: therefore, we set $F_r^{{\alpha }{\beta }}=F_r$ for any ${\alpha },{\beta }\in \left\{ N,P\right\}$. Regarding cell-cell adhesion, we assume that N-cadherin–N-cadherin bonds generate symmetric adhesive responses in each of the adjacent cells, i.e., we take $F_a^{NP}=F_a^{PN}=F_a^{NN}$, since both cell lines express N-cadherin molecules. PCs additionally express E-cadherin, thereby generating further strong and stable homotypic bonds. Consequently, it is reasonable to take $F_a^{PP}>F_a^{NN}=F_a^{NP}=F_a^{PN}$.

Beyond these biologically motivated considerations, the estimates of $F_r$ and $F_a^{{\alpha }{\beta }}$ must still be treated with care. Specifically, regions of the parameter space exist that can generate a physically unrealistic system evolution, such as “population collapse” in which the individuals condense into an infeasibly close aggregate. Consequently, the set of interaction coefficients must be chosen to ensure that a relaxed configuration can be attained and maintained, characterized by fixed and finite mutual distances: a so-called *crystalline* pattern. In this respect, it has been shown that the large-time behaviour of particle systems subjected to non-local pairwise interactions is related to the H-stability of the relative kernels and potentials, see Ruelle (1969). In particular, taking advantage of the characterization of H-stable potentials provided in Ruelle (1969), recent works (such as Cañizo et al. 2015; Cañizo and Patacchini 2018; Carrillo et al. 2018) provide a criterion that determines a subregion of the parameter space of the repulsive-adhesive interacting kernels that results in realistic crystalline cell pattern. Accounting for these analytical results, for any pair $\alpha \beta \in \{NN, NP, PN, PP\}$, the parameters $F_r$ and $F_a^{{\alpha }{\beta }}$ must satisfy the relation

13 $$\begin{aligned} \dfrac{F_r}{F_a^{{\alpha }{\beta }}}>\frac{4\,d_a^3}{5\,d_r^3}. \end{aligned}$$

For the above specified cell sizes, this becomes $F_r/F_a^{{\alpha }{\beta }}>21.6$. Despite its usefulness in preventing unrealistic situations, this criterion still does not indicate the exact pairing of interaction parameters. In this respect it is then useful to remark that cells are unable to move too rapidly, i.e., $|{\mathbf {v}}_{\text {res}}^{{\alpha }}(t) + {\mathbf {v}}_{\text {adh}}^{{\alpha }}(t)|$ should not exceed reasonable values (e.g., $v_{\text {max}}$). Preliminary simulations where we switch off all velocity components apart from the interaction terms are therefore performed to highlight the role of cell-cell repulsion/adhesion on system behaviour, for the purposes of parameter estimation.

**Fig. 4:**
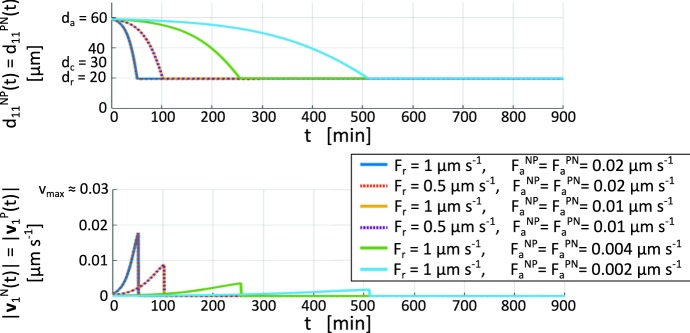
Estimate of the heterotypic cell-cell interaction parameters. Evolution of the relative distance and the speed of two cells, of type N and P respectively, initially located at a distance equal to $59~\upmu \hbox {m}$, with dynamics regulated by only the interaction velocity component. It is possible to observe that the system behaviour is robust with respect to variations in $F_r$, but sensitive to variations in $F_a^{NP}=F_a^{PN}$, which set the time needed to establish crystalline configurations. Note that the blue and dashed red curves, as well as the yellow and dashed purple curves, are indistinguishable (color figure online)

Specifically, we first consider a two cell system composed of a NC and a PC, in order to focus on heterotypic cell-cell interactions. The NC individual is placed at $(200~\upmu \hbox {m},350~\upmu \hbox {m})$ and the PC at $(259~\upmu \hbox {m},350~\upmu \hbox {m})$, i.e., such that $d^{NP}(0)$ is fractionally below $d_a$. The time evolution of the system is evaluated through $d_{11}^{NP}(t)$ and the speeds $|{\mathbf {v}}_{1}^{N}(t)|=|{\mathbf {v}}_{1}^{P}(t)|$, which are equal for any $t\in [0,T_{\max }]$ as a consequence of our assumption of symmetric pairwise interactions. The results in Fig. 4 show that the behaviour does not change significantly with variations in $F_r$: see the overlap between the first and second curves, or between the third and fourth. Conversely, the system is highly sensitive to variations of $F_a^{NP}=F_a^{PN}$, which define the timescale required to establish a crystalline configuration in the multicellular case. We further remark that cell speeds remain plausible under all considered parameter settings.

**Fig. 5:**
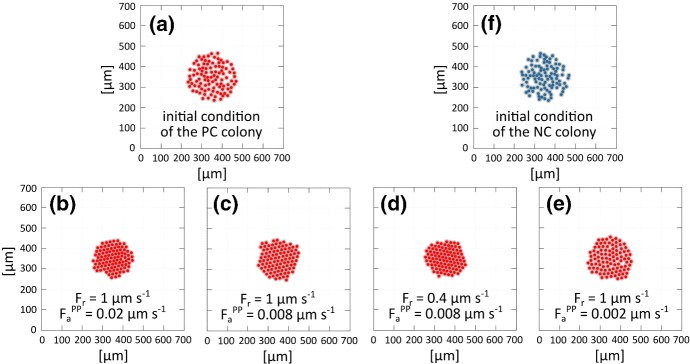
Estimate of the homotypic cell–cell interaction parameter. PC cluster behaviour under different parameter settings, each satisfying the H-stability condition given in Eq. (13). **a** Initial distribution of the PC colony used for all numerical simulations. **b**–**e** Cell distribution after approximately 2 h. **f** Initial distribution of the NC colony used to estimate the relative homotypic interaction parameters. In all panels, cells are represented by circles of diameter $d_r$ to highlight how the minimal intercellular distance and intrinsic resistance is large enough to prevent significant overlap. This representation will be used in all subsequent figures

We next turn to the role of homotypic cell-cell interaction parameters on model outcomes. We consider the behaviour of a colony of 100 PCs for three different cell-cell interaction coefficient parameter settings, each satisfying the H-stability condition: $F_r=1~\upmu \mathrm {m\,s}^{-1}$ with $F_a^{PP} = 0.02~\upmu \mathrm {m\,s}^{-1}$; $F_r=1~\upmu \hbox {m}\,\hbox {s}^{-1}$ with $F_a^{PP} = 0.008~\upmu \mathrm {m\,s}^{-1}$; $F_r=0.4~\upmu \mathrm {m\,s}^{-1}$ with $F_a^{PP} = 0.008~\upmu \mathrm {m\,s}^{-1}$; and $F_r=1\,\mu \mathrm {m\,s}^{-1}$ with $F_a^{PP} = 0.002~\upmu \mathrm {m\,s}^{-1}$. All simulations start from the same initial condition: an almost round cluster of radius $\approx 120~\upmu \hbox {m}$ placed in the centre of the domain, see Fig. 5a. To focus on adhesive/repulsive stimuli, we recall that we switch off the random velocity component (i.e., ${\mathbf {v}}^P_{j,\text {rand}}(t)={\mathbf {0}}$, for any $j=1,\dots ,n_P$ with $t\in [0,T_{\text {max}}]$) and note that the CIL velocity components will be zero for each PC (i.e., ${\mathbf {v}}^P_{j,\text {CIL}}={\mathbf {0}}$ for any $j=1,\dots ,n_P$ with $t\in [0,T_{\text {max}}]$), due to the absence of NCs. As shown in Fig. 5b–e, in all cases the PC colony generates and maintains a crystalline configuration (without collapse or dispersion), consistent with our parameter selection within the H-stability region of the system.

On fixing $F_r$, decreasing $F_a^{PP}$ (see Fig. 5b–c, e) generates a more dispersed colony, since reducing adhesive contributions allows further emergence of repulsive effects. The most dispersed configuration we can obtain under adhesive-repulsive interactions alone arises by setting $F_a^{PP} = 0~\upmu \mathrm {m\,s}^{-1}$, and is characterised by bounded minimal interparticle distances that do not trespass $d_r$ due to the definition of $f_{\text {res}}^{\alpha \beta }$ in Eq. (4) (see Cañizo et al. 2015; Cañizo and Patacchini 2018; Carrillo et al. 2018 and references therein for further details). On the other hand, on fixing $F_a^{PP}$ we observe that decreasing $F_r$ results in a slight decrease of the equilibrium interparticle mean distance, specifically towards a highly packed colony (with a small degree of cell membrane overlap, see Fig. 5c, d). However, by considering parameter pairs that satisfy the H-stability condition in Eq. (13), a minimal interparticle distance is preserved and finite. It is still necessary to control whether selected parameters pairs generate interparticle distances not significantly lower than $d_r$. In this respect, in Fig. 5 cells are represented by circles of diameter $d_r$ to highlight that a sensible minimal intercellular distance is maintained, and we keep such a representation in subsequent figures to demonstrate this important property. Given these experiments, we set $F_a^{PP}=0.008~\upmu \mathrm {m\,s}^{-1}$ and $F_r = 1~\upmu \mathrm {m\,s}^{-1}$ (Fig. 5c): for consistency with experimental observations, we require the maintenance of a highly packed PC colony, but without cell overlap.

The same study is then performed for the NC aggregate, starting from an equivalent initial condition (Fig. 5f). Fixing $F_r = 1~\upmu \mathrm {m\,s}^{-1}$ (as for the PCs), from our preliminary simulations we set $F_a^{NN} = 0.002~\upmu \mathrm {m\,s}^{-1}$ to generate a more dispersed NC aggregate with respect to the PC cluster (compare panels (c) and (e) in Fig. 5), in accordance to *in vitro* experiments in Theveneau et al. (2013). According to the assumption that $F_a^{NN} = F_a^{NP} = F_a^{PN}$, we finally set $F_a^{NP} = F_a^{PN} = 0.002~\upmu \mathrm {m\,s}^{-1}$.

**Fig. 6:**
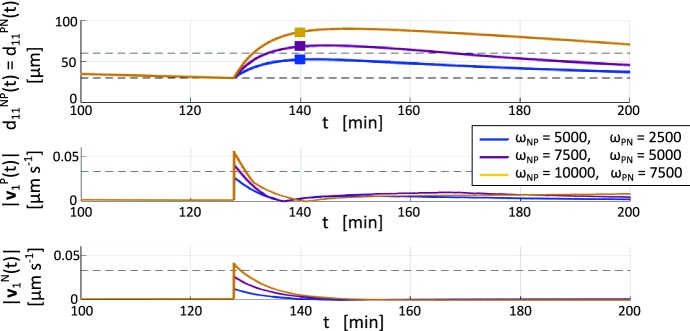
Estimates of the parameters characterising the CIL velocity components. Cell intercellular distance $d_{11}^{NP}(t)=d_{11}^{PN}(t)$ and speed of the two interacting individuals in three parameter settings: i.e., $\omega _{NP} = 5000$ with $\omega _{PN}=2500$ (blue case); $\omega _{NP} = 7500$ with $\omega _{PN}=5000$ (purple case); and $\omega _{NP} = 10{,}000$ with $\omega _{PN}=7500$ (yellow case). Squares in the top panel indicate the value of $d_{\text {CIL}}$ for each case (color figure online)

*Parameters related to CIL velocity components* For realistic estimates of the parameters $\tau _{{\alpha }{\beta }}$ and $\omega _{{\alpha }{\beta }}$, with ${\alpha }{\beta }\in \left\{ NP,PN\right\}$, that characterise the CIL velocity, we start by considering the experimental literature (see, among others, Fig. 6 (b) in Theveneau et al. 2013 for heterotypic CIL responses). In particular, regardless of cell phenotype, we assume that the Rac1-dependent intracellular cascade that is responsible for the CIL mechanism is activated for some given time such that $\tau _{NP} = \tau _{PN} = 6\ \text {min}$, since we estimate the overall CIL contribution to last $\approx 12\ \text {min}$. Then, accounting for images in Theveneau et al. (2013) relative, as seen, to heterotypic dynamics, we can assume that the instantaneous push resulting from CIL is dictated by the intrinsic migratory abilities of the re-polarized cell: since NCs are known to have more significant motility than PCs, we therefore state $\omega _{NP} > \omega _{PN}$.

To estimate their values, we analyse how a two-cell system composed of a single neural crest and a single placode cell evolves upon variations of $\omega _{NP}$ and $\omega _{PN}$. To highlight CIL velocity components we switch off the random term while maintaining other parameters as previously estimated (and listed in Table 1). Further, for an accurate fit between the numeric and experimental measures in Scarpa et al. (2013) and Theveneau et al. (2013), we use the following critical quantities for comparison: (i) the cell mutual distance evaluated $12~\mathrm {min}$ post NC-PC collision (hereafter denoted by $d_{\text {CIL}}$); and (ii) the maximum velocity reached by the two individuals as a consequence of CIL (hereafter denoted by $v_{\text {CIL}}$). From the data generated by the biological experiments, we can assume that reasonable values for $\omega _{NP}$ and $\omega _{PN}$ should result in $d_{\text {CIL}}\approx 50~\upmu \hbox {m}$ and $v_{\text {CIL}}<3~\upmu \mathrm {m\, min^{-1}} = 0.05~\upmu \mathrm {m\,s}^{-1}$. From the plot of Fig. 6 we observe that such empirical data can be reasonably matched by setting $\omega _{NP} = 5000$ and $\omega _{PN}=2500$.

**Fig. 7:**
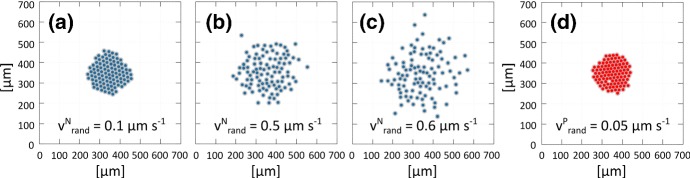
Estimate of parameters related to random velocity components. **a**–**c** Final configuration (i.e., at $t=300~\mathrm {min}$) of the NC colony for different settings of $v^{N}_{\text {rand}}$. **d** Final configuration (i.e., at $t=300~\mathrm {min}$) of the PC colony for the selected value of $v^{P}_{\text {rand}}$

*Parameters related to random velocity components* We first recall from Eq. (11) that $\theta _{N}(t)$ and $\theta _{P}(t)$ are random variables uniformly distributed on $[0, 2 \pi )$. The values of $v^{N}_{\text {rand}}$ and $v^{P}_{\text {rand}}$ instead denote the intensity of Brownian fluctuations. Analysing the experimental literature (see, among others, Fig. 2(a, b) of Theveneau et al. 2013), we can observe that NCs have significantly greater autonomous intrinsic motility (i.e., isotropic oscillations) than PCs. Hence we set $v^{N}_{\text {rand}} \gg v^{P}_{\text {rand}}$ and perform a series of simulations, starting from the same initial conditions used to estimate homotypic interaction parameters (see Figs. 5a, f). In all cases, cell dynamics are regulated only by homotypic interactions and random velocity contributions and all other contributions are subsequently neglected.

Simulation results under variations of $v^{N}_{\text {rand}}$ (Fig. 7a–c) are compared against their experimental counterpart: the best-fit (measured in terms of cell displacement and intercellular distance) emerges for $v^{N}_{\text {rand}} = 0.6~\upmu \mathrm {m\,s}^{-1}$. The dynamics of placode cell clusters are highly determined by the strong homotypic E-cadherin interactions that maintain cluster shape, and we observe little change upon alteration of $v^{P}_{\text {rand}}$. We therefore choose a value $v^{P}_{\text {rand}} = 0.05~\upmu \mathrm {m\,s}^{-1}$ and remark that simulations are relatively insensitive against variations in this parameter.

### Two-cell system

**Fig. 8:**
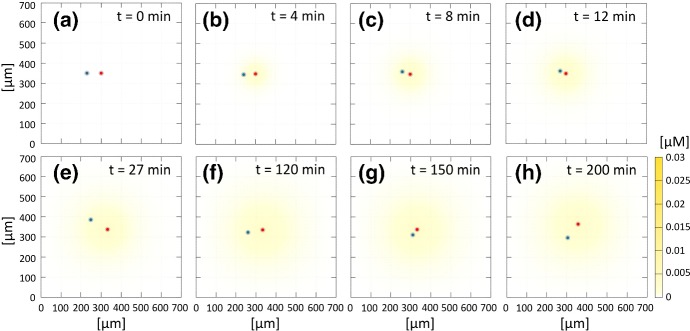
Time-lapse of the two-cell system evolution in the case of the reference simulation. **a** Initial position of NC (blue) and PC (red). **b**–**h** Representative frames showing cell dynamics that characterise the “chase-and-run” process. The colour-coded bar indicates Sdf1 concentration. A movie of this simulation is included as Supplementary Movie M1 (color figure online)

**Fig. 9:**
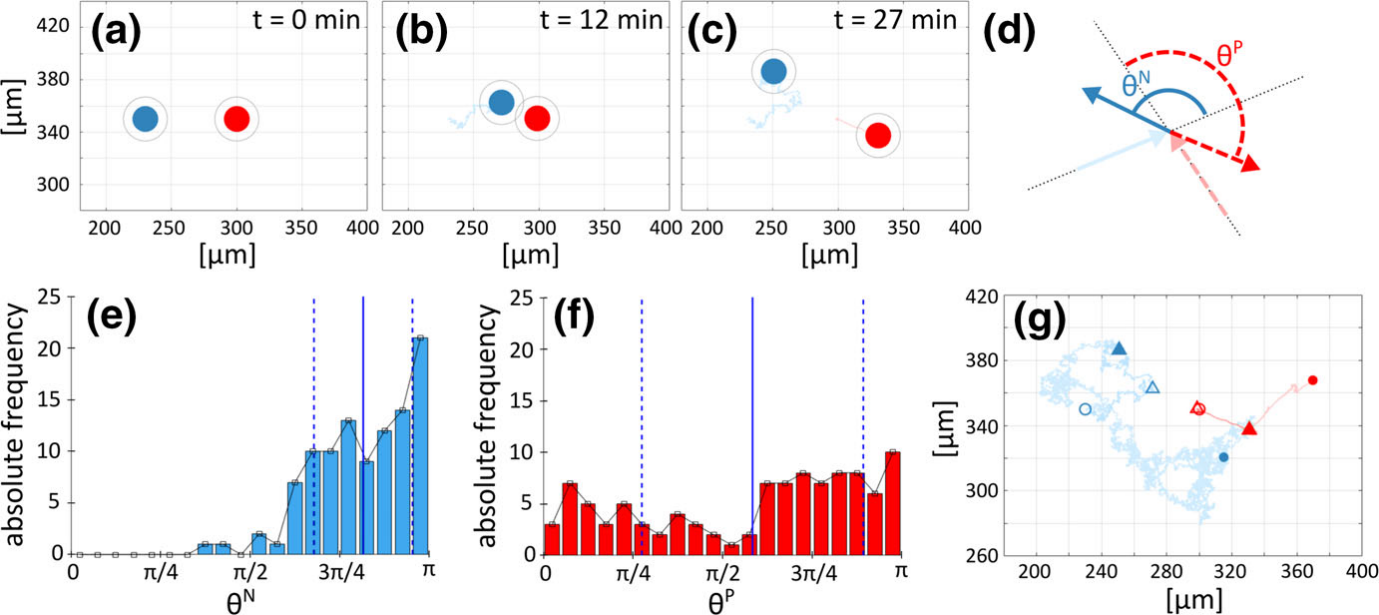
“Chase-and-run” dynamics in the reference simulation. **a**–**c** Blue and red circles and lines represent positions and trajectories over [0, *t*] for the NC and PC, respectively. The diameter of the solid circles reflects $d_r$, while outer circles represent $d_c$: we highlight how CIL is triggered as the intercellular distance drops below $d_c$. Note that, for clarity of presentation, we select only a central portion of the overall domain. **d** Representation of angles $\theta ^N$ (solid blue angle) and $\theta ^P$ (dashed red angle) for cell direction of motion before and after an NC-PC contact activates the CIL response. The solid light blue and dashed pink arrows represent, respectively, NC and PC direction of motion before contact, i.e., $\mathbf{x }_i^{\alpha }(t_c-\varDelta t)/|\mathbf{x }_i^{\alpha }(t_c-\varDelta t)|$ with $\alpha \in \{N,P\}$, where $t_c$ denotes the instantaneous time of the cell-cell collision and $\varDelta t=9~\mathrm {min}$ as in Theveneau et al. (2013). Analogously, the solid blue and dashed red arrows indicate NC and PC direction of motion after contact, i.e., $\mathbf{x }_i^{\alpha }(t_c-\varDelta t )/|\mathbf{x }_i^{\alpha }(t_c-\varDelta t )|$. **e**–**f** Absolute frequency following 100 numerical simulations for the angle identifying NC (left) and PC (central) velocities before and after the first NC-PC collision. The solid blue line gives the mean, whereas dashed blue lines indicate the variance. **g** Trajectory of NC (blue) and PC (red) cell over an entire simulation (5 h). Empty and filled circles denote the initial and final positions of NC (blue) and PC (red), respectively; empty triangles indicate cell positions at the point of the first NC-PC collision in this realisation and filled triangles mark their positions 15 min after collision (color figure online)

**Fig. 10:**
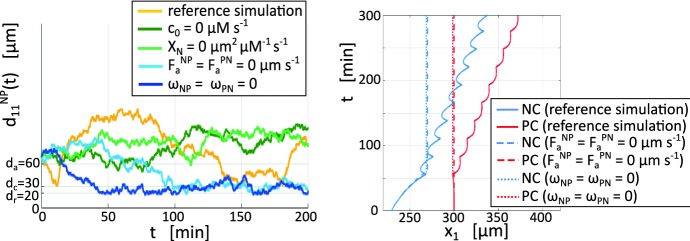
Left panel: evolution in time of cell relative distance $d_{11}^{NP}(t)$ in the reference simulation (yellow curve) and under disruptions of chemotaxis (green curves), N-cadherin bond formation (light blue curve) and intracellular CIL response (dark blue curve). Right panel: evolution in time of the *x*-coordinate for the cell positions (i.e., $\mathbf{x}^N_i(t)$ and $\mathbf{x}^P_i(t)$, respectively) in the reference simulation (full lines) and under disruptions of N-cadherin bond formation (dashed lines) and intracellular CIL response (light dashed lines); for these specific simulations, the random contributions to individual movement are neglected (color figure online)

We begin with a two-cell system, i.e., based on the interactions between a single NC and a single PC. We first perform a reference simulation that highlights “normal” dynamics, before replicating the impact of selected experimental disruptions. In each simulation, we initially space the cells $70~\upmu \hbox {m}$ apart: in this respect, $\mathbf{x }^{N}_1(0)=(230~\upmu \hbox {m}, 350~\upmu \hbox {m})$ and $\mathbf{x }^{P}_1(0)=(300~\upmu \hbox {m}, 350~\upmu \hbox {m})$ define the cell starting locations, see Fig. 8a and Fig. 9a. To assess the evolution of the system under various scenarios, our primary measurement will be the *intercellular distance*, $d_{11}^{NP}$, which is defined as the distance between the point positions marking cell centres and is reported in the left panel of Fig. 10.

*Reference simulation* As observed from time-lapse images in Fig. 8 (and from Supplementary Movie M1), random wandering dominates until the Sdf1 field forming around the PC reaches the NC. Subsequently, the NC chemotactically migrates towards the PC, generating the chase phase of the process (see Fig. 8c). As the intercellular distance drops below $d_a$, adhesive interactions occur and the two individuals further approach each other, with their mutual distance narrowing accordingly (Fig. 8d and yellow curve of Fig. 10). When the intercellular distance drops to $d_c$, CIL is triggered (see Fig. 9b and Fig. 10). CIL then induces the NC and PC to “bounce” away one from each other (Fig. 8e and Fig. 9c), moving apart until quasi-stabilising at a distance dictated by the magnitude of the CIL response (Fig. 9c).

A return to the initial Brownian motion is then observed and the show plays on repeat, see Fig. 8f–h, with chemotactic-driven chase drawing cells together until CIL induces a further bounce.

The post-contact directionality of the cells, shown in Fig. 9d–f, lies in qualitative agreement with the experimental literature (see, for instance, Fig. 6 (c) of Theveneau et al. 2013). Specifically, in Theveneau et al. (2013) angles were calculated using three positions for each of the NC and PC, corresponding to $9~\mathrm {min}$ before a contact, at a contact, and $9~\mathrm {min}$ after a contact. Changes in the direction of motion of the NC and PC, following CIL activation, are thus quantified here by the angles $\theta ^{\alpha }$ (with $\alpha \in \{N,P\}$) represented in Fig. 9d, where the solid light blue and dashed pink arrows denote, respectively, the NC and PC direction of motion before contact, while the solid blue and dashed red arrow indicate NC and PC direction of motion after contact. Notice that the measure $\theta ^{\alpha }$ and the approach used to calculate the corresponding angle measured in Theveneau et al. (2013), for $\alpha \in \{N,P\}$, are consistent. To account for the random component to cell dynamics, means and variances of $\theta ^N$ and $\theta ^P$ are inferred following 100 realisations of the reference test, see Fig. 9e–f, and the resulting values are consistent with experimental measurements reported in Theveneau et al. (2013).

Overall, there is a net directional cell migration, as captured by the substantial shift in the cell positions from the beginning to end of the observation time, see Fig. 9g. However, the global displacement is strongly affected by the random velocity component, as indicated by the fluctuations in $d_{11}^{NP}(t)$ (see the yellow curve in the left panel of Fig. 10). For the sake of completeness, cell paths along the *x*-axis are evaluated in the absence of Brownian fluctuations: as demonstrated in the right panel of Fig. 10, chemotaxis promotes the early directional movement of the NC individual, whereas a coordinated net locomotion of the two cells is obtained only in the presence of a fully active CIL response.

**Fig. 11:**
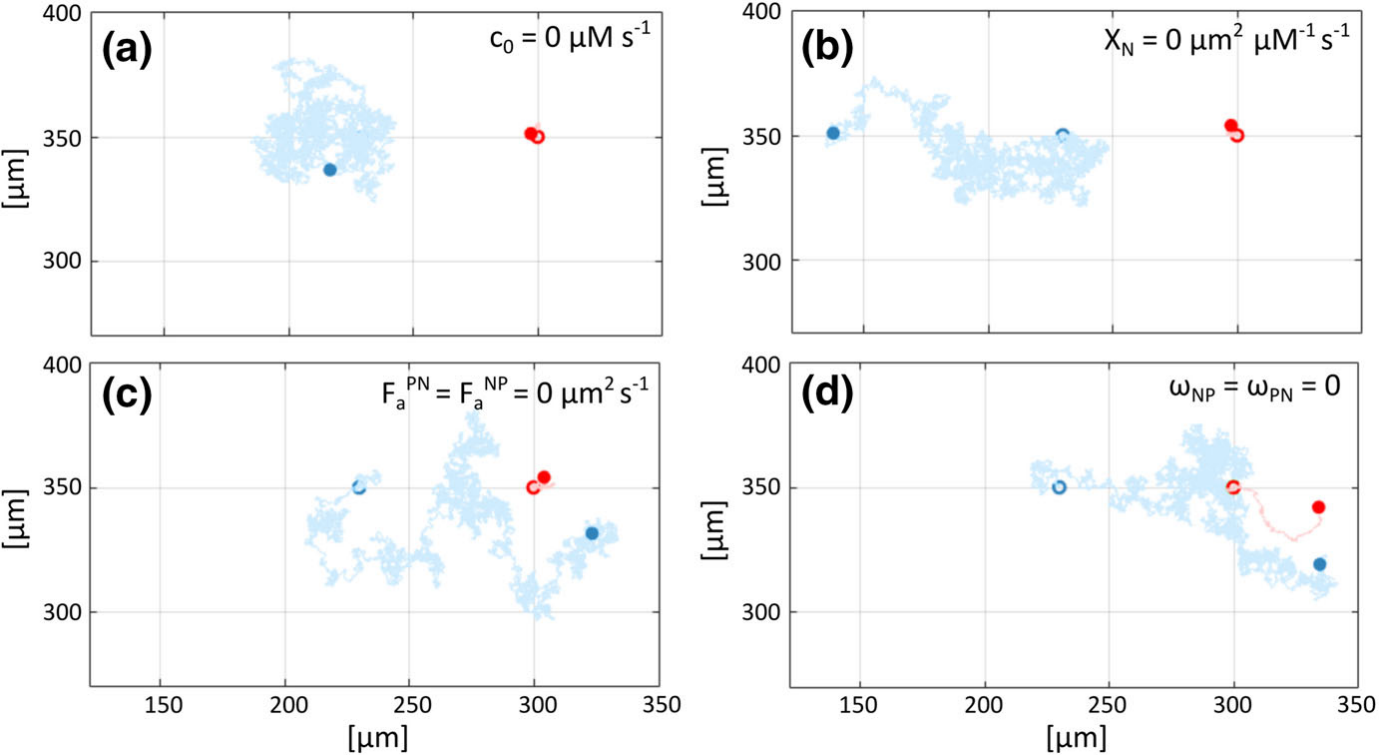
Two-cell system behaviour upon selected disruptions of model components. In all images, NC and PC trajectories are respectively depicted in blue and red. The empty and filled circles indicate the initial and final cell positions, respectively. **a** Inhibition of Sdf1 production by PC (i.e., $c_0=0~\upmu \mathrm {M\, s^{-1}}$ in Eq. (12)). **b** Disruption of NC chemotactic response (i.e., $\chi _{N}=0~\upmu \mathrm {m^2}~\upmu \mathrm {M^{-1} s^{-1}}$ in Eq. (9)). **c** Inhibition of N-cadherin bond formation [i.e., $F_a^{NP} = F_a^{PN} = 0~\upmu \mathrm {m\,s}^{-1}$ in Eq. (6)]. **d** Direct disruption of CIL response [i.e., $\omega _{NP} = \omega _{PN} = 0$ in Eq. (7)]. In all cases, other velocity terms and relevant parameters are set as in the reference simulation (color figure online)

*Disrupting NC chemotaxis migration* We next target the capacity of the NC to chemotactically migrate, by either (i) inhibiting the Sdf1 production from the PC individuals (setting $c_0=0~\upmu \hbox {M}\,\hbox {s}^{-1}$ in Eq. (12)), or (ii) switching off the chemotactic sensitivity term (setting $\chi _{N}=0~\upmu \mathrm {m^2~\upmu M^{-1} s^{-1}}$ in Eq. (9)), corresponding to a knock-out of Cxcr4 receptor activity. In both cases (see Fig. 11a, b), the NC is unable to sense the PC and therefore the two cells exhibit uncorrelated random crawling about their initial positions: the intercellular distance hovers about (and can also exceed) its initial value over the simulation timecourse (light and dark green lines in the left panel of Fig. 10). We note that crawling is more pronounced for the NC individual, as expected given that the choice $v^{P}_{\text {rand}}\ll v^{N}_{\text {rand}}$ in Eq. (11) leads to $|{\mathbf {v}}^{P}_{1,\text {rand}}|\ll |{\mathbf {v}}^{N}_{1,\text {rand}}|$ for any $t\in {\mathbb {R}}_+$. Note that while random wandering can potentially bring cells within contact range, and hence induce CIL, this is not typically observed within the simulation timecourse.

*Disrupting N-cadherin bonds* We then inhibit N-cadherin adhesive bond formation through setting to null the heterotypic adhesive strengths, i.e., $F_a^{NP} = F_a^{PN} = 0~\upmu \mathrm {m\,s}^{-1}$ in Eqs. (6)–(7). This disruption acts to shut down adhesive and CIL velocity contributions, thus ${\mathbf {v}}^{N}_{1,\text {adh}}(t) = {\mathbf {v}}^{P}_{1,\text {adh}}(t) = {\mathbf {v}}^{N}_{1,\text {CIL}}(t)={\mathbf {v}}^{P}_{1,\text {CIL}}(t)= {\mathbf {0}}$ for all $t \in [0, T_{\text {max}}]$ in Eq. (1). It is worth remarking that, in this case, the inhibition of CIL arises through a block in the activity of the N-cadherin complexes, which initiate the intracellular Rac1-dependent cascade that culminates in the CIL response (see hp 2 in Sect. 2). Typical trajectories are shown in Fig. 11c and we chart the intercellular distance and the directional cell displacement in the left panel of Fig. 10 (light blue curve). We observe that the chemotactic-driven chase occurs as normal, with the two cells stabilising at a distance $d_{11}^{NP}(t)\in [d_r,\,d_a]$. The subsequent run phenomenology is no longer obtained and disruption of the adhesive bonds allows cells to approach closer than $d_c = 30~\upmu \hbox {m}$ without bouncing back. This lies in contrast to the reference case, where normal chase behaviour is maintained only for intercellular distances $d_{11}^{NP} (t) \ge d_c$ and below this threshold the run phase is invoked. These observations lie in accordance with biological data (see, for instance, Fig. 6 (b) in Theveneau et al. 2013). Fluctuations in intercellular distance are now mainly dictated by the compression-driven repulsive velocity contributions, which enter when the intercellular distance falls below $d_r = 20~\upmu \hbox {m}$. Note that the small perturbations in cell positions stem again from the random velocity components.

*Disrupting the CIL response* Our final disruption targets directly the intracellular cascade responsible for CIL, setting $\omega _{NP} = \omega _{PN} = 0$ in Eq. (7). Consequently, only CIL velocity contributions are neglected, i.e., ${\mathbf {v}}^{NP}_{1,\text {CIL}}(t) = {\mathbf {v}}^{PN}_{1, \text {CIL}} (t)= {\mathbf {0}}$ for all $t \in [0, T_{\text {max}}]$. In particular, we remark that adhesive interactions still occur through the formation of N-cadherin adhesive bonds. Resulting dynamics are captured by cell trajectories (see Fig. 11d) and by the time evolution of intercellular distances (dark blue curve in left panel of Fig. 10). As for disruption of N-cadherin adhesive bonds, the two cells now stabilise at distances $d_{11}^{NP}(t)\in [d_r,\,d_a]$ without bouncing back. This highlights how an inhibition acting directly and only on the Rac1-dependent CIL pathways (i.e., without affecting the activity of the N-cadherin molecules) is sufficient to disrupt normal “chase-and-run” behaviour, again in noteworthy agreement with the corresponding experiments (Theveneau et al. 2013). A subtle distinction arises in cell behaviour due to disruption of either the Rac1-dependent intracellular cascade or the N-cadherin adhesive bonds, however, and it can be captured by quantifying the magnitude of intercellular distance fluctuations. Specifically, we have smaller fluctuations of $d_{11}^{NP}(t)$ in the former scenario due to the normal activity of adhesive interactions for $d_{11}^{NP}(t)<d_a$, which continue to exert a degree of control over random and repulsive velocity contributions.

### Multicellular populations system

**Fig. 12:**
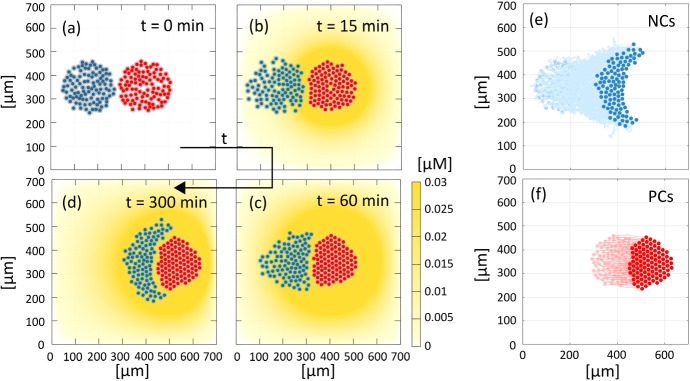
Reference simulation of the multicellular system. **a**–**d** Evolution of the complete model with 100 NCs (left cluster, in blue) and 100 PCs (right cluster, in red). The color-coded bar indicates Sdf1 concentration. A movie of this simulation is included as Supplementary Movie M2. **e**–**f** Trajectories of NCs and PCs that highlight overall net collective and coordinated migration (color figure online)

We next explore “chase-and-run” collective behaviour in a multicellular system, formed by a population of neural crest cells that interact with a placode aggregate. As above, we first consider a reference simulation for the complete model to highlight quantitative and qualitative determinants of the coordinated cell movement. We subsequently describe system phenomenology under selected mechanistic disruptions. For all experiments we consider 200 cells, composed of equal numbers of NC and PCs (i.e., $n_N = n_P = 100$), with the same initial set-up: a culture formed from two adjacent aggregates, one of NCs and one of PCs, each arranged in a quasi-round cluster of radius $\approx 120~\upmu \hbox {m}$, see Fig. 12a. This initial configuration is similar to the experimental counterpart in Theveneau et al. (2013).

As critical measures to quantify system behaviour, we will take the minimal homotypic and heterotypic distances between the generic *i*-th cell belonging to population $\alpha \in \{N, P\}$ and the other individuals, i.e., $d^{{\alpha }{\beta }}_{i, \text {min}}= \min _{j=1,\ldots ,n_{{\beta }}}d^{{\alpha }{\beta }}_{ij}$, with ${\beta }\in \{N, P\}$.

*Reference simulation* As illustrated in Fig. 12 (and in Supplementary Movie M2), neural crest cells initially undergo collective chemotaxis in the direction of the placode colony, maintaining a quite compact configuration (which mainly stems from the common directional velocity rather than from the low homotypic adhesive interactions). As NC cells at the leading edge of the cluster approach the PC cluster, heterotypic CIL responses are initiated and PC individuals subsequently move away from the NCs, see Fig. 12c. Reverse movements of NC cells, however, are blunted by their ongoing chemotactic responses and by the adhesive interactions (albeit low). As a result, we observe a consistent and collective directional motion of the entire system: the PC cluster is gradually shunted towards the right, pushed and pursued by the NC aggregate, see Fig. 12d. The collective cell phenomenology captured by our simulations (summarised by the trajectories in Fig. 12e–f) lie in general accordance with corresponding *in vitro* experimental dynamics (cf. Fig. 2(c)–(h) in Theveneau et al. 2013), although we remark that in our numerical simulations the chasing NC cluster becomes increasingly crescent-like in shape. As we show below, the conformation of the NC aggregate subtlety changes upon parameter variation and we will further return to this aspect in the discussion.

**Fig. 13:**
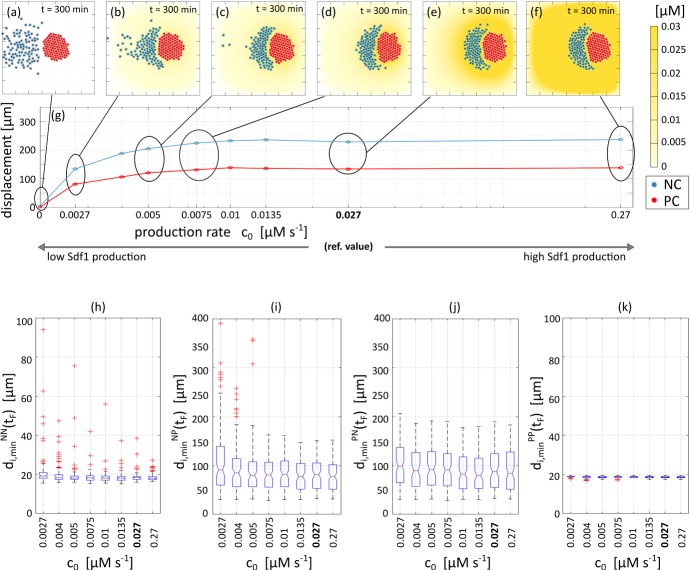
Effect of variable Sdf1 production by the PCs. **a**–**f** Final configurations, i.e., at $t=300~\mathrm {min}$. **g** Displacements of the centres of mass for the two aggregates: the reference value $c_0 = 0.027~\upmu \mathrm {M\,s^{-1}}$ used in Fig. 12 is written in bold type. **h**–**k** The boxplots report the minimal intercellular distance between cells, from left to right: **h** between a NC and other NCs; **i** between a NC and the PCs; **j** between a PC and the NCs; and, **k** between a PC and other PCs

*Disrupting NC chemotaxis migration* Our first virtual knock-out focuses on NC chemotactic movement. We first vary Sdf1 production by the PCs, i.e., we vary $$c_0$$ in Eq. (12). Given sufficiently high secretion rates of the chemical ($$\ge 0.01$$$$~\upmu \mathrm {M\,s^{-1}}$$), the NC aggregate forms a compact aggregate that chases the PCs, as in the reference scenario, see Fig. 13d–f. For lower values of $$c_0$$ (i.e., $$< 0.01$$$$~\upmu \mathrm {M\,s^{-1}}$$), however, the trailing region of the NC colony instead scatters, see Fig. 13a–c. In this case a comet-like tail forms from individual cells that have lost contact with the main mass, due to not receiving a sufficiently high stimulus of the diffusing chemoattractant. In such cases, the collective directional movement of the system is slightly downregulated, probably as a consequence of the lower pushing force that the dispersed NC cluster exerts on the PC island. Notably, however, the formation of such tails can be observed in experiments (cf. Fig. 2 (c) in Theveneau et al. (2013) and its accompanying movies). Finally, and consistent with the two-cell scenario, complete inhibition of Sdf1 production (i.e., $$c_0= 0$$$$~\upmu \mathrm {M\,s^{-1}}$$) leads to abolition of chase-and-run and the NC and PC cells/colonies crawl about their initial position, as shown in Fig. 13a and in the first column of Fig. 16. Here, NCs (as a whole) do not perceive the PCs and subsequently move randomly, independently with respect to the PCs (with the exception of those initially located close to the PC cluster such that random wandering brings them within adhesive range).

Addressing Fig. 13h–k, it can be observed that the production of $c_0$ does not influence homotypic placode intercellular distances, since $d^{NN}_{i, \text {min}}$ and $d^{PP}_{i, \text {min}}$ are predominantly regulated by adhesion. Neither is the heterotypic distance $d^{PN}_{i, \text {min}}$, since (excluding the complete knock out case) there are always NC cells that reach the PC aggregate, see Fig. 13j. The heterotypic distance $d^{NP}_{i, \text {min}}$, however, presents a more scattered distribution, with a significant number of outliers emerging for low values of $c_0$, see Fig. 13i. This phenomenology accompanies the above described trail and results from single cells that have detached from the main NC aggregate, subsequently subjected to greater degrees of random migration.

**Fig. 14:**
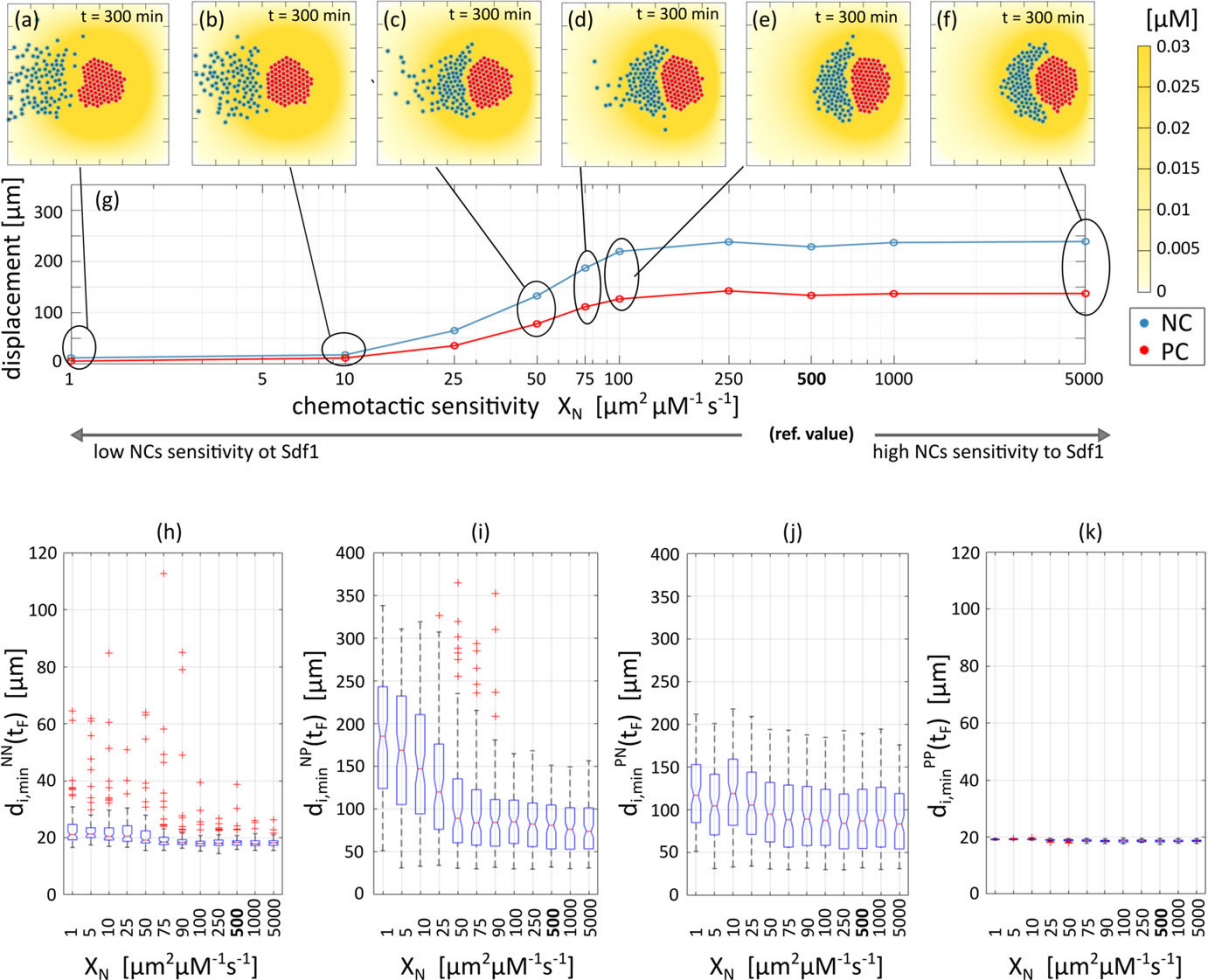
Effect of variable Sdf1 sensitivity by the NCs. **a**–**f** Final configurations, i.e., at $t=300~\mathrm {min}$. **g** Displacements of the centres of mass for the two aggregates: the reference value $\chi _N = 500~\upmu \mathrm {m^2~\upmu M^{-1}\,s^{-1}}$ used in Fig. 12 is written in bold type. **h**–**k** Boxplots report the minimal intercellular distance between cells, from left to right: **h** between a NC and other NCs; **i** between a NC and PCs; **j** between a PC and NCs; and, **k** between a PC and other PCs

We now keep Sdf1 production constant and vary instead the expression/activity of NC Sdf1 receptors (i.e., varying $$\chi _N$$ in Eq. (9)). As shown in Fig. 14f, high values of $$\chi _N$$ (i.e., $$> 100$$$$\mathrm {~\upmu m^2\, ~\upmu M^{-1} s^{-1}}$$) result in the compact chemotactic migration of the NC cluster and CIL-driven coordinated net displacement of the two populations, as in the reference case. A slight downregulation of the NC chemical response (i.e., $$\chi _N \in [50, 100]$$$$\upmu \mathrm {m^2\, ~\upmu M^{-1} s^{-1}}$$) instead promotes dispersion of NCs into the trailing region of the cluster and significant inhibition of the run phase of the process, see Fig. 14c–e. Significantly low values of $$\chi _N$$ (say $$\lesssim 25$$$$\upmu \mathrm {m^2\, ~\upmu M^{-1} s^{-1}}$$) completely disrupt NC chemotactic movement and there is negligible overall directional locomotion of the system, see Fig. 14a–d. As highlighted by the first two columns of Fig. 16, complete knock-out of cell sensitivity has effectively the same impact on cell trajectories as a complete knock-out of Sdf1 production. Boxplots for these investigations are similar to those observed following Sdf1 production variation, compare Fig. 13h–k and Fig. 14h–k. We do remark, however, that there is a clearer dependence of the heterotypic minimal intercellular distance between an NC and PCs (see $$d_{i\text {min}}^{PN}(t_F)$$ in Fig. 14j), with outliers present only in the transition region when the NC trail forms.

**Fig. 15:**
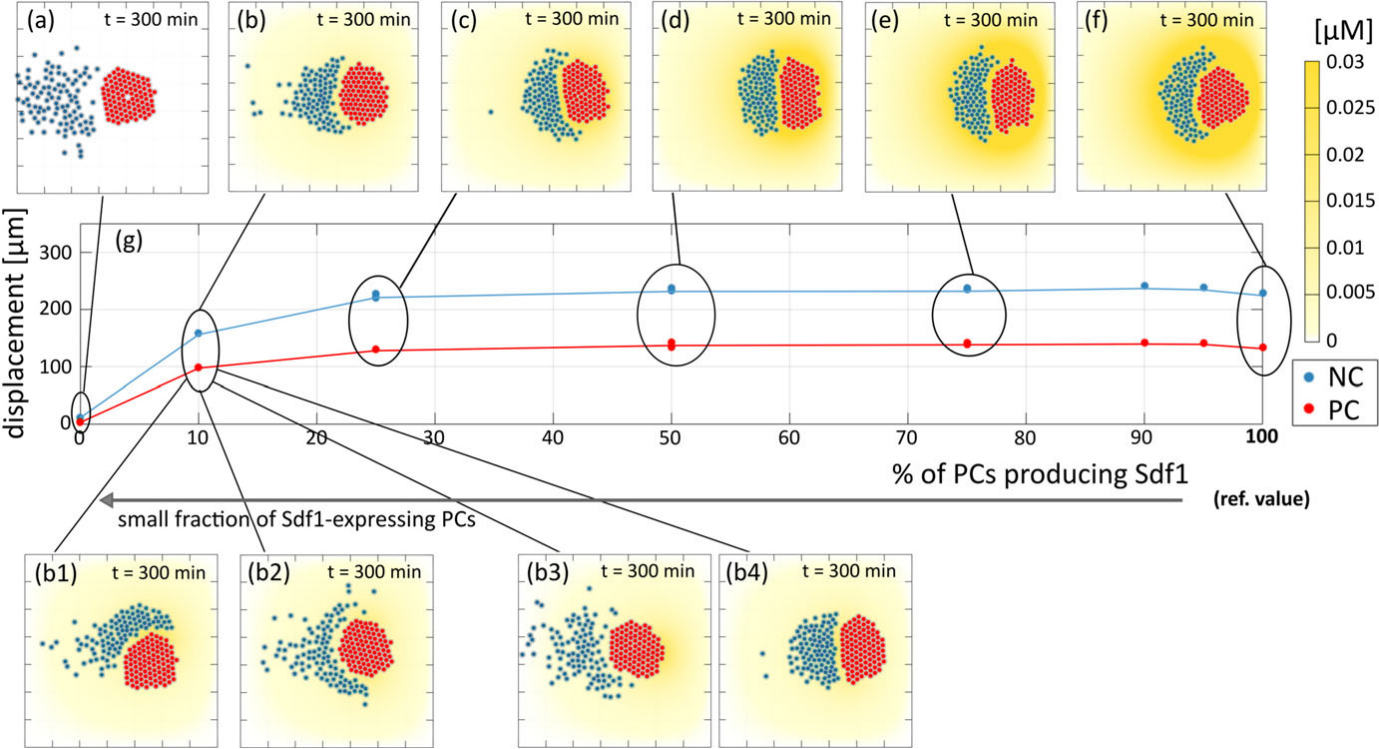
Effect of blocking Sdf1 production in a given fraction of the PC population. **a**–**f**, **b1**–**b4** Final configurations, i.e., at $t=300~\mathrm {min}$. In particular, **a** corresponds to the complete knock-out of Sdf1 production in all cells, whereas **f** is the reference case reported in Fig. 12 where all PCs secrete Sdf1. **g** Displacement of the center of mass of the two aggregates. **b1**–**b4** Final configurations under specific localisation of the ($10\%$) PCs that produce Sdf1: **b1**$10\%$ positioned within the north edge of the PC colony; **b2**$5\%$ positioned within each of the north and south edges; **b3**$10\%$ positioned within the east edge; and **b4**$10\%$ positioned within the west edge

We finally predict the effect of completely blocking Sdf1 production, but only in a given fraction of the placode population. As shown by Fig. 15c–f, chase and run behaviour (as measured by the group displacement) is relatively unaffected under moderate disruptions but becomes significantly reduced when only few placode cells secrete the chemical substance (i.e., $\lesssim 10\%$), see Fig. 15a, b. It is interesting to note that reducing the number of PCs producing Sdf1 also generates a morphological transition in the NC aggregate, which morphs from a “crescent moon” shape encircling the PCs to an ellipsoid configuration adjacent to the PC cluster (see Fig. 15f) and, finally, a more dispersed cluster accompanied by the comet-tail (see Fig. 15a).

For the simulations reported in Fig. 15a–f, Sdf1 expressing PCs are randomly positioned in the cluster. We next investigate how their localisation within the PC colony impacts on the chase-and-run phenomenon. Specifically, in Fig. 15b1–b4 we consider four distinct cases for the scenario under which only $10\%$ of the PC population produces Sdf1. In Fig. 15b1, all Sdf1-expressing cells are placed within the north edge of the PC colony; in Fig. 15b2, half are placed within the north edge of the PC colony and the remainder within the south edge; in Fig. 15b3, all Sdf1-expressing PCs are within the east edge of the group; and, in Fig. 15b4, all are positioned within the west edge of the PC aggregate. Under the first scenario, the induced asymmetrical pattern of Sdf1 results in NCs moving towards the northern edge of the PC colony, disrupting both the symmetric comet-tail distribution shown in Fig. 15b and the displacement of the PC colony. In Fig. 15b2, NCs organise into a crescent moon that envelopes the PC colony, being attracted towards both the north and the south edges of the aggregate. This results in a slight reduction of net displacement (with respect to Fig. 15b, where PCs producing Sdf1 are randomly distributed). The comet-tail configuration observed in Fig. 15b is preserved in the other cases (Fig. 15b3–b4) but there are distinct changes to the net migration. For Sdf1-expressing PCs concentrated at the eastern edge, the final distribution is similar to that obtained in Fig. 15a, i.e., in the absence of Sdf1 producing cells, and there is little net migration: NCs are distant from the source of Sdf1 production and minimal chemotactic migration/chase-and-run ensues. On the other hand, see Fig. 15b4, localising the secreting PC population to the west edge allows NCs to organize into a compact aggregate that stimulates chase-and-run, with a displacement comparable to the levels observed in Fig. 15b–c. Thus, while precise positioning of the secreting cells may act to significantly disrupt chase-and-run/cell displacement, any benefits (in terms of increasing the net displamcent) from optimal localisation are marginal (in respect to the randomly distributed case of Fig. 15b). Moreover, we remark that the importance of localisation within the PC colony decreases as the fraction of Sdf1 producing PCs increases.

**Fig. 16:**
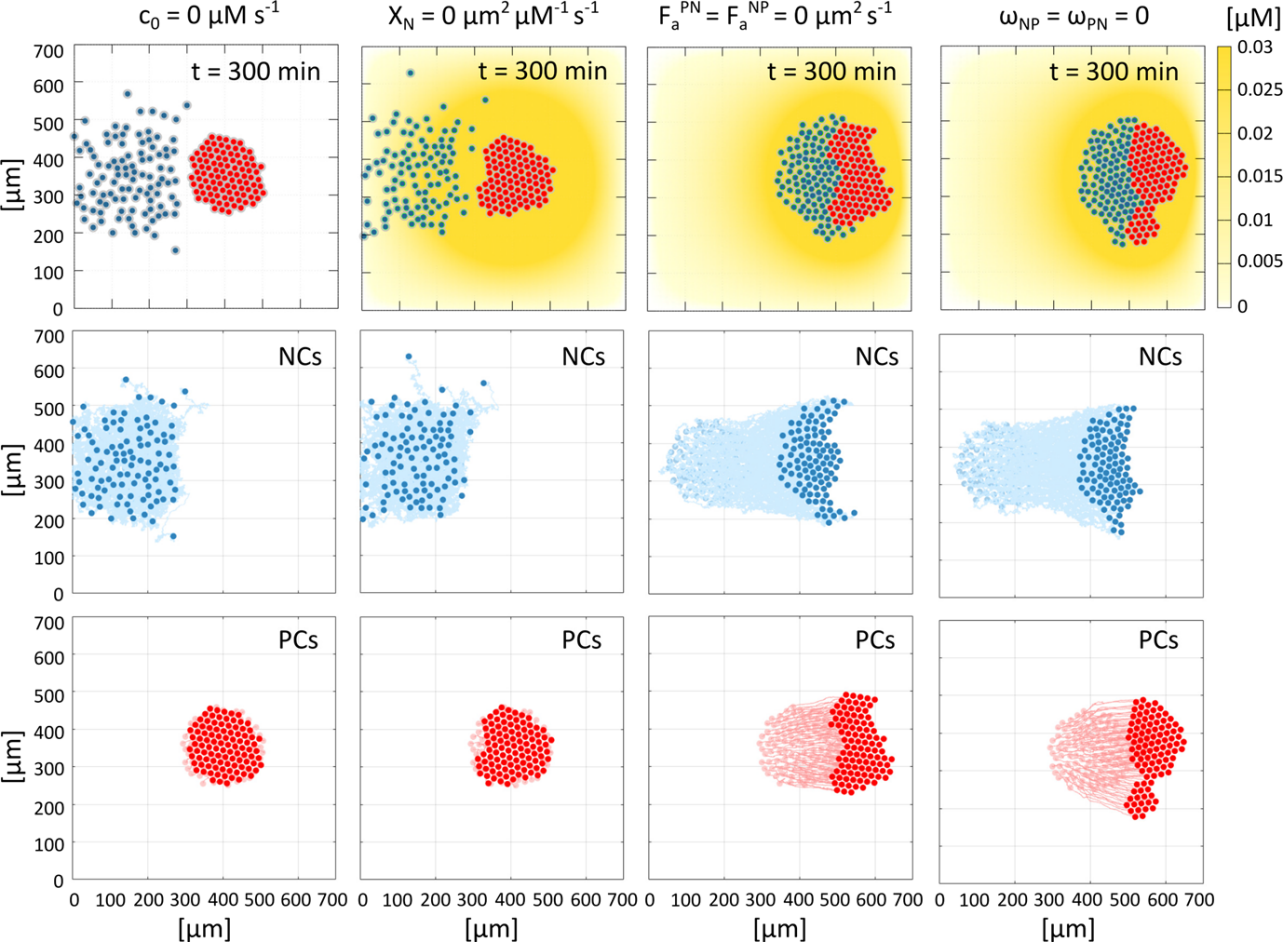
Final configuration, i.e., at $t=300~\mathrm {min}$, of NC and PC colonies and cell trajectories in the case of complete knock-outs, namely: inhibition of Sdf1 production by the PCs (leftmost column); inhibition of expression/activity of NC Sdf1 receptors (second column); inhibition of N-cadherin adhesive bond formation (third column); and, disruptions of the Rac1 dependent intracellular cascade responsible for the CIL response (rightmost column)

*Disrupting N-cadherin bonds* We next inhibit the formation of N-cadherin bonds, setting adhesive strengths $F_a^{NN} = F_a^{NP} = F_a^{PN} = 0~\upmu \mathrm {m\,s}^{-1}$ while keeping $F_a^{PP} = 0.008~\upmu \mathrm {m\,s}^{-1}$ in Eq. (5) and in Eq. (7). The non-zero PC-PC adhesive parameter stems from their additional expression of E-cadherin. Consequently, we have ${\mathbf {v}}^{NN}_{i,\text {adh}}(t) = {\mathbf {v}}^{NP}_{i,\text {adh}}(t) = {\mathbf {v}}^{PN}_{j,\text {adh}}(t)={\mathbf {0}}$, as well as ${\mathbf {v}}^{NP}_{i,\text {CIL}}(t) = {\mathbf {v}}^{PN}_{j,\text {CIL}}(t)={\mathbf {0}}$ with $i=1,\dots ,n_N$ and $j=1,\dots ,n_P$, for any $t\in [0,T_{\text {max}}]$. Other velocity components and parameters remain the same as the reference simulation. As observed in the third column of Fig. 16 (see also Supplementary Movie M3), the NC aggregate continues to move via chemotaxis towards the PCs; the two populations subsequently form a single quasi-round cluster, with a central region characterised by mixing of the two cell lineages. The absence of N-cadherin interactions precludes activation of a CIL response and disrupts the “run” phase of the dynamics. This lies in qualitative agreement with the corresponding biological experiment (cf. Fig. 6 (j) in Theveneau et al. (2013)). However, we remark that the *in vitro* setting has a three-dimensional element where, despite the 2D plating, a degree of cell overlap occurs at the interface between the two aggregates. This aspect is precluded by our numerical realisation, a consequence of the strictly two-dimensional domain used as a simplification.

*Disrupting the CIL response* We next target the intracellular cascade responsible for the CIL response, i.e., we set $\omega _{NP} = \omega _{PN} = 0$ in Eq. (7). Hence, ${\mathbf {v}}^{NP}_{i,\text {CIL}}(t) = {\mathbf {v}}^{PN}_{j,\text {CIL}}(t) = {\mathbf {0}}$ with $i=1,\dots ,n_N$ and $j=1,\dots ,n_P$, for any $t\in [0,T_{\text {max}}]$, while the other velocity components/parameters remain as the reference simulation. The system behaviour, captured in the last column of Fig. 16, is analogous to the case of disruption of N-cadherin bonds. Chemotaxis allows the NC aggregate to chase the PC aggregate, as above, and the two populations form a single aggregate in a similar manner to the previous setting, and in qualitative agreement with biological experiments (e.g., see Fig. 6 (j) in Theveneau et al. 2013).

**Fig. 17:**
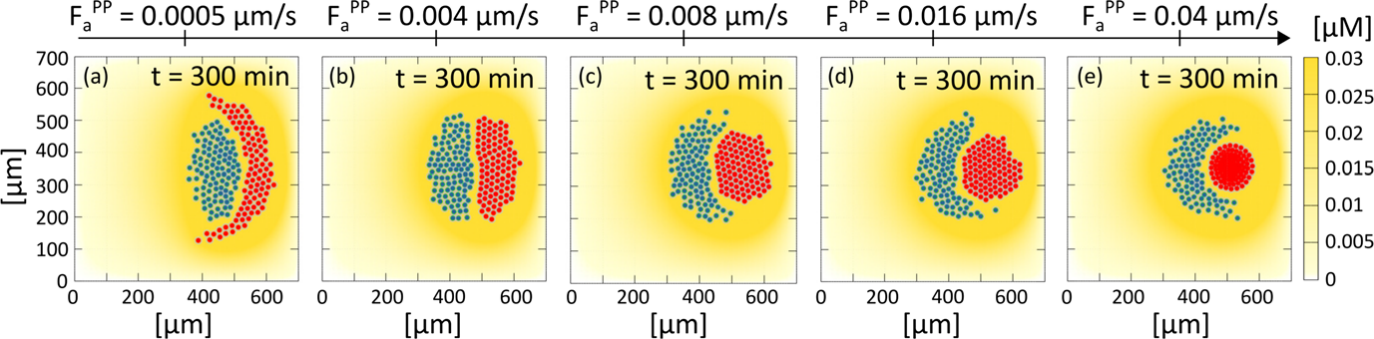
Final configurations, i.e., at $t=300~\mathrm {min}$, of the clusters following variation in the expression/activity of E-cadherin molecules, which regulate PC homotypic adhesiveness and are quantified by the model parameter $F_a^{PP}$. As a remark, other adhesion parameters are set at $F_a^{NN} = F_a^{NP}= F_a^{PN} = 0.002\, ~\upmu \hbox {m}\, \hbox {s}^{-1}$

*Variation in PC E-cadherin expression* We again use our approach in a predictive manner. In particular, we test system dynamics upon variations in the PC E-cadherin expression/activity (evaluated by parameter $F_a^{PP}$), which, as far as we are aware, has not been systematically explored from an experimental perspective. As in previous perturbation-type experiments, all other model components and coefficients are kept according to the reference case setting. As shown in Fig. 17, PC homotypic adhesiveness strictly correlates with the morphological characteristics of the system. For large values of $F_a^{PP}$ (i.e., $\gg F_a^{NN}=F_a^{NP}=F_a^{PN} = 0.002\, ~\upmu \hbox {m}\, \hbox {s}^{-1}$), the PC cluster behaves in the manner of a quasi-rigid disk and becomes surrounded by the NC aggregate, see Fig. 17c–e. As PC homotypic adhesion is lowered to a similar magnitude to other adhesion contact strengths (see Fig. 17b) both cell clusters undergo coordinated movement while maintaining a quasi-elliptical shape. Finally, when placode self adhesion becomes substantially reduced (i.e., to the point that $F_a^{PP} \ll F_a^{NN}=F_a^{NP}=F_a^{PN} = 0.002\, ~\upmu \hbox {m}\, \hbox {s}^{-1}$), we observe a capacity of the NC population to infiltrate and disperse the PC cluster into an encapsulating crescent-like cluster, see Fig. 17a. We further remark that within the simulation timescales, net directional migration of the system is not significantly affected by variations to PC homotypic adhesiveness.

**Fig. 18:**
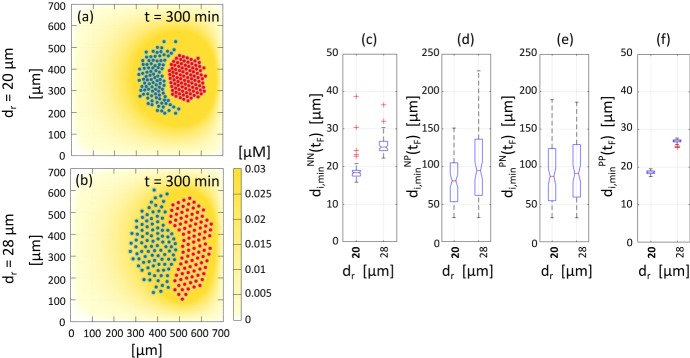
Effect of changing the repulsive distance. **a**, **b** final configurations, i.e., at $t=300~\mathrm {min}$, with distances of the order of the nucleus and of the cell diameters. **c**–**f** boxplots of the minimal intercellular distances

*Variation in the extension of the range of repulsive dynamics* We finally provide predictions on how cell dynamics are altered following variation of cell repulsive behaviour. In particular, an increment in the value of $d_r$ can be viewed as a numerical counterpart to an increment in the stiffness of the cytosolic region that surrounds the cell nucleus. The cell cytoskeleton is, in fact, formed by a contractile acto-myosin cortex whose rigidity can be modulated through chemical manipulation. As expected, and highlighted in Fig. 18a, b, under larger values of $d_r$ the homotypic clusters become more extended/dispersed. As revealed in Fig. 18c, f, the minimal homotypic distances $d^{NN}_{i, \text {min}}$ and $d^{PP}_{i, \text {min}}$ are strongly correlated with $d_r$. On the other hand, focusing on Fig. 18d, e, the heterotypic distances $d^{NP}_{i, \text {min}}$ and $d^{PN}_{i, \text {min}}$ (effectively measuring the distance between the two cell aggregates) are almost insensitive to variations in $d_r$. This arises from the fact that heterotypic repulsion is, rather, predominantly regulated by CIL, which does not allow NC individuals to become too close to PCs. We note that again, in this case, no other changes have been made to model components from the reference case scenario.

## Discussion

We have explored “chase-and-run” dynamics in heterogeneous cell populations. Specifically, we have encoded a computational model for the evolution of a system composed of plated neural crest and placode cells. Chemotaxis of NCs in response to a placode-secreted attractant triggers the chase phase and a subsequent CIL response generates the run phase of the process. By limiting to a purposefully small set of assumptions and replicating the *in vitro* set-up of two populations initially placed in juxtaposing aggregates, our computational simulations have been able to reproduce the experimental findings in Theveneau et al. (2013) in semi-quantitative fashion, i.e., to capture the productive net migration of the cell clusters. Yet, some deficiencies of this minimalist approach are raised by our simulations. We use this discussion to further elaborate, and discuss subsequent extensions to be considered in future modelling.

**Fig. 19:**
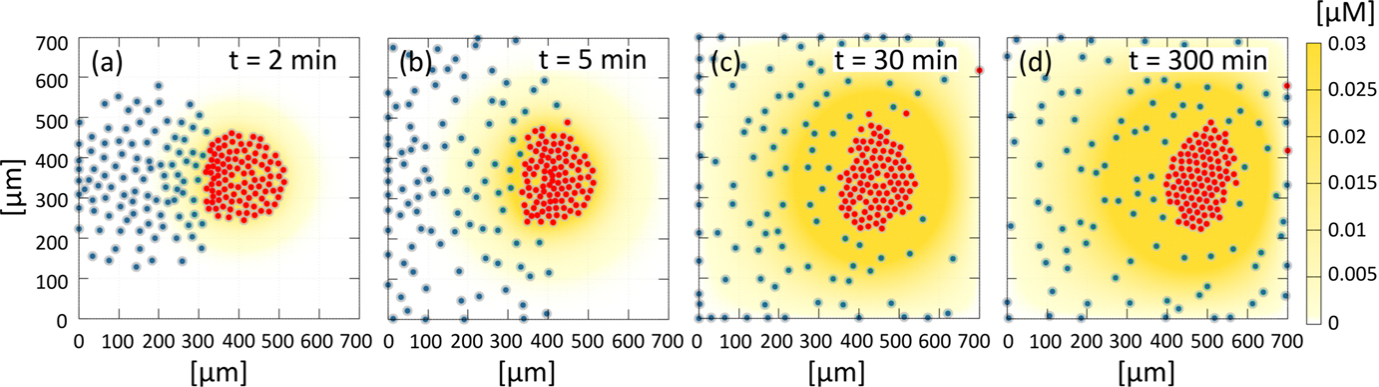
Numerical simulation with homotypic CIL between NCs, highlighting the excessive scattering of the NC colony with respect to experimental observations. In this case, $\tau _{NN}=\tau _{NP}$ and $\omega _{NN}=\omega _{NP}$)

First, we have taken a simplistic approach to the inclusion of NC-NC homotypic interactions. In the context of CIL, since neural crest cells express N-cadherins one should naturally consider that collisions between two migrating NCs will also generate mutual CIL responses (Carmona-Fontaine et al. 2008). Homotypic CIL interactions between two NC cells^1^ can be trivially incorporated in a manner equivalent to the corresponding heterotypic ones: the first equation in Eq. (1) can be augmented with an additional velocity component, say ${\mathbf {v}}_{i,\,\text {CIL}}^{NN}$, whose dynamics are governed by essentially the same law as for the contribution due to heterotypic NC-PC interactions (i.e., ${\mathbf {v}}_{i,\,\text {CIL}}^{NP}$, defined in Eq. (7)), but now generated through NC-NC interactions. Simulations analogous to those of Sect. 3.3, see the representative case plotted in Fig. 19, though, highlight a subsequently poor agreement with *in vitro* outcomes: when homotypic CIL interactions are explicitly incorporated, excessive scattering of the NC colony occurs and “chase-and-run” is clearly impaired.

Experimental and theoretical studies (Camley et al. 2016a, b; Carmona-Fontaine et al. 2011; Merchant et al. 2018; Szabo et al. 2016; Woods et al. 2014) support an additional NC-NC co-attraction mechanism, in which their secretion of an attracting substance (complement fragment C3a) counterbalances the effects of CIL-induced dispersal and maintains cohesion of the NC cluster. In the interests of model simplicity we have excluded explicit incorporation of this mechanism at present – explicit inclusion would demand an extra equation for the additional molecular species and an accompanying increase in the dimensionality of the parameter space. Rather, this co-attraction has been implicitly included, naively by assuming it counteracts the dispersal effects from homotypic CIL. Of course, an important development for future studies would be explicitly extending the model to account for this and determining in turn how it impacts on chase-and-run dynamics.

We further remark on the crescent-like shape of our chasing NC cluster in the reference simulation (see Fig. 12), which is somewhat different from the rounder aggregate observed in experimental controls, e.g. in Theveneau et al. (2013). Notably, NC crescents are observed under certain experiments scenarios, for example under partial inhibition of CIL (cf. second panel of Supplementary Movie 14 in Theveneau et al. 2013) or when clusters are initially separated by a greater distance (cf. Fig. 2 (h) in Theveneau et al. 2013), suggesting that crescent shapes are by no means unnatural features of the *in vitro* system. This phenomenon arises naturally from the model mechanism, where chemotaxis allows cells at the back of the NC aggregate to gradually work their way around the group and accumulate at the NC-PC interface. This crescent-like shape becomes more pronounced as the simulation proceeds (cf. Fig. 12 and its accompanying Supplementary Movie, M2, where earlier snapshots show a more circular geometry for the NC cluster), possibly suggesting that our simulations artificially accelerate this process. Notably, we have observed a tendency towards a reduction in the degree of crescent formation as certain parameters are shifted: for example, lower values of PC homotypic adhesion, reductions in the percentage of PCs that secrete Sdf1 and increments in the extension of the intercellular repulsive region. However, a crescent or ellipsoidal shape is not entirely eradicated, suggesting that further mechanisms may act to maintain the round shape of the NC cluster. Of course, a possible candidate lies in explicitly including the above mentioned coattraction mechanism, rather than the naive implicit inclusion here, or through some form of group polarisation/coordination within the NC cluster.

Both one-to-one and many-to-many simulations indicate that “chase-and-run” can generate an overall productive net cell migration, with NCs and PCs significantly shifting from their initial locations. However, the directionality is substantially different in the distinct settings. In one-to-one experiments random motility plays a fundamental role as the orientation of the PC, with respect to the NC, deviates significantly after the CIL response (and compare the axial direction connecting NC to PC in their starting and final locations, see Fig. 9g). In contrast, for cell clusters the Brownian crawling effects are diminished and net movement is predominantly along the axial direction connecting their initial centres of mass. In this respect, we can in principle claim that efficient movement along a specific direction demands cluster to cluster interactions rather than one cell to one cell interplay. Of course, directionality is strongly dependent on the initial placement of the cell aggregates and current simulations only cover distances of the order of 100 $\mu m$: *in vivo*, NC cells may need to travel significantly further and it is unclear whether the present mechanism would allow precise persistent movement without further guidance signals, such as long range chemical attractants or specific substrate patterns.

Parametrisation is a key issue for computational/mathematical models in the biosciences. Ideally, parameters could be obtained directly from biological data and, where possible, we have attempted to source such values (see Table 1). However, even if a parameter is estimated directly from its data, its reliability is questionable if its originates from distinct experimental studies, different developmental processes or from other species. Other parameters may have no clear biological analogue and here our approach has been to estimate such parameters through fitting to biological data (e.g. comparing characteristic run lengths). Building further details of molecular binding events and their subsequent impact on movement via inhibiting/promoting focal adhesion formation would potentially allow closer connection to quantitative data, although clearly such an approach would increase both the complexity of the model and the dimensionality of the parameter set. The principal aims of the current study are more exploratory in nature: employing a “top-down” modelling approach to test the fundamental hypothesis that a relatively minimal set of interactions can capture the biological observations of “chase-and-run”. Nevertheless, further insights into the criticality and sensitivity of parameters is desirable and in this regard a more comprehensive sensitive analysis and further analytical investigation would be important.

For practical purposes we have targeted a specific cell system, where model hypotheses can be constrained. Nevertheless, the overall framework is generic and can be adapted to address other systems where similar mechanisms are believed to operate, e.g., in the case of cancer cell populations (Astin et al. 2010) or of developmental macrophages (Stramer et al. 2010). Here, contact-based interactions are mediated through cadherins and quasi-identical responses are assumed in the contacting cells. For other contact-based cell dynamics, such as under Eph/Ephrin signalling (Kania and Klein 2016) or in zebrafish pigment cells (Yamanaka and Kondo 2014), the reaction of each individual involved may be markedly different, involving attraction in the former case and repulsion in the latter. Thus, contact-based interactions can lead to a variety of homotypic (i.e. between cells of the same type) and heterotypic (between cells of different type) movement responses and a theoretical study into how various combinations act to control patterned collective movements of heterogeneous tissue populations would be of keen interest. In this respect, we refer to Painter et al. (2015) for a study using a fully continuous model.

While explicit analytical explorations are often difficult in agent-based models, we remark on two areas that could benefit from further mathematical exploration. First, we have built on recent investigations on the H-stability properties of adhesive/repulsive pairwise interaction potentials (Cañizo et al. 2015; Cañizo and Patacchini 2018; Carrillo et al. 2018; Ruelle 1969), that have allowed us to identify parameter regimes under which the model system evolves to a physically realistic configuration. Such analytical studies have in fact offered us welcome constraints for streamlining the tricky process of parametrisation. Second, we note that the *chase time* required for a neural crest cell to contact a placode cell (and subsequently initiate CIL) can be interpreted in the context of a mean first passage time problem (e.g., see McKenzie et al. 2009; Redner 2001): the average time needed by some object (in this case, the neural crest cell) to hit a target (the placode cell). A detailed exploration would require extension of existing theory, for example to account for the moving nature of the target and the fact that the bias is driven by chemotaxis. While we defer to a future investigation, such studies could shed light on, for example, how the chase phase is determined by the diffusive and reaction kinetics of the attractant.

The approach proposed in this work has extended previous modelling studies on CIL via an explicit study into how such a mechanism, coupled to other processes, drives the collective migration of heterogeneous interacting cell systems. Further, our computational framework has incorporated an explicit dynamical equation that governs the strength and the duration of the CIL response for each cell (see Eq. (7)). While our parametrisation has been guided here through an experimental-matching procedure, our model is open to future refinements: for example, connecting these parameters to an explicit representation of the intracellular signalling that regulates the CIL response. Such model developments would offer a potential pathway to targeted *in silico* predictions on the impact in cell behaviour of molecular-level perturbations.

## Electronic supplementary material

Below is the link to the electronic supplementary material.

Supplementary material 1 (mp4 1147 KB)

Supplementary material 2 (mp4 7225 KB)

## Footnotes

1 PCs also express N-cadherin, although any subsequent CIL response between two PCs is expected to be negated due to the overriding action of E-cadherin binding.
